# Temporal and geographic distribution of gut microbial enterotypes associated with host thermogenesis characteristics in plateau pikas

**DOI:** 10.1128/spectrum.00020-23

**Published:** 2023-10-10

**Authors:** Xianjiang Tang, Liangzhi Zhang, Shi'en Ren, Yaqi Zhao, Yanming Zhang

**Affiliations:** 1 Key Laboratory of Adaptation and Evolution of Plateau Biota, Northwest Institute of Plateau Biology, Chinese Academy of Sciences, Xining, China; 2 Qinghai Provincial Key Laboratory of Animal Ecological Genomics, Xining, China; 3 University of Chinese Academy of Sciences, College of Life Sciences, Beijing, China; Brigham Young University, Provo, Utah, USA

**Keywords:** enterotype, plateau pika, spatiotemporal dynamics, thermogenesis, community assembly

## Abstract

Differentiation of the gut microbiota between individuals within a species can be identified by enterotypes. However, whether different enterotypes are associated with host-related physiological status and adaptation to specific habitats is still poorly understood. In this study, we collected 308 cecal samples from plateau pikas during varying seasons and altitudes. We identified three bacterial and two fungal enterotypes. The proportion of *Rikenellaceae_RC9_gut_group*- and *Prevotella*-predominated enterotype 3 was higher than that of the other two enterotypes in all samples. Enterotype 1, distinguished by norank *Muribaculaceae*, was mainly detected during the warm season and at high altitudes, whereas enterotype 2, which is unclassified *Lachnospiraceae*, was mainly detected during the cold season. Plateau pikas with bacterial enterotypes 2 and 3 showed significantly higher alpha diversity than those with enterotype 1, while pikas with fungal enterotype 1 displayed higher diversity than those with enterotype 2. Functional pathways of lipid metabolism were enriched in bacterial enterotype 1, carbohydrate and amino acid metabolism were enriched in enterotype 2, and energy metabolism was enriched in enterotype 3. The neutral process was more important in bacterial enterotype 3 than in enterotypes 1 and 2. The body temperature and resting metabolic rate of pikas with bacterial enterotypes 1 and 3 were significantly higher than those with enterotype 2. Pikas with unclassified *Sporormiaceae*- and *Sporormiella*-dominated fungal enterotypes may engage more frequently in coprophagy. Overall, this study revealed that both gut bacterial and fungal communities in pikas can be clustered into enterotypes, and individuals with different enterotypes showed significant differences in thermogenesis.

**IMPORTANCE:**

The gut microbiotas of small mammals play an important role in host energy homeostasis. However, it is still unknown whether small mammals with different enterotypes show differences in thermogenesis characteristics. Our study confirmed that plateau pikas with different bacterial enterotypes harbored distinct thermogenesis capabilities and employed various strategies against cold environments. Additionally, we also found that pikas with different fungal enterotypes may display differences in coprophagy.

## INTRODUCTION

Although the gut microbiota has a high species identity, inter-individual variation is also large (1, 2). This variation is mainly attributed to differences in host genetic and physiological status, such as age, sex, and reproduction, as well as lifestyle or habitat environment (3 – 7). To identify this distinction, a relatively new clustering analysis based on the Calinski-Harabasz (CH) index, called enterotype, was used to describe individuals with different groups of gut microbiota (8 – 10). The differences in enterotypes are driven by groups of species that together contribute to the preferred community composition (8 – 10). Research suggests that the human gut microbiome can be partitioned into three enterotypes: *Bacteroides*, *Prevotella*, and *Ruminococcus*, respectively (8). Studies on gut microbiota in chimpanzees, pigs, buffalo, mice, and honeybees also detected enterotypes, which indicated that enterotypes are probably ubiquitous in animal gut microbiota (11 – 15). A previous study regarding the human oral cavity microbiome using large samples also found two major “stomatotypes,” driven by *Neisseria* and *Prevotella* (16), which suggested that the new clustering analysis can also be applied to bacterial communities from different habitats as well as different microbial taxa.

The gut microbiome is a longstanding co-evolutionary symbionts with the host (17). It has been proved that gut bacteria play an important role in host nutrition fermentation, especially for dietary fiber digestion, energy metabolism, physiology, and health (18 – 21). Although fungi occupy less than 1% of the gut microbiome (22), it is also pivotal in host nutrition, metabolic health, and immune development (23 – 26). Variation in the gut microbiome is usually associated with functional variations; therefore, individuals harboring gut microbiome variations indicate their distinct lifestyle or dietary habits (27 – 30). Additionally, individuals harboring distinct gut microbiomes may exhibit distinct physiological statuses, cognitive competences, or athletic abilities (31 – 33). For example, studies on the human enterotype suggested that individuals who are engaged in diets rich in protein and animal fat usually possess a *Bacteroides*-dominant enterotype, whereas the *Prevotella*-dominant enterotype is usually found in individuals with a ﬁber-rich diet (10). Additionally, a previous study on the gut microbiota of athletes showed that individuals with different enterotypes displayed significant differences in athletic performance (33). Enterotypes are usually thought to be relatively stable and unique for a stable host lifestyle or physiological status (10, 33). However, recent studies have suggested that enterotypes display deterministic transitions with host diet, physiological status, and living environment variation (9, 11, 14, 34). For example, a high-fat and protein diet fed to mice housed in a laboratory environment reduced the enterotype category compared with that of wild mice, and the enterotypes began to mimic those identified in humans (14). To some extent, plant secondary compounds can restore these changes to the wild state (9). However, it is unclear whether the enterotypes are closely associated with host lifestyle or physiological status, as well as the category of enterotypes, or changes with seasons and habitats.

Plateau pika (*Ochotona curzoiae*) is a non-hibernating small herbivorous mammal that is widely distributed in the Qinghai-Tibet Plateau at altitudes of 3,100–5,200 m (35 – 37). The plateau pika is considered a pest due to their destruction of grasslands (38). However, increasing evidence has demonstrated that plateau pika is a keystone species in the Qinghai-Tibet Plateau owing to its key functional traits (37). A previous study on plateau pika samples from different seasons at one sample site suggested that plateau pika possessed three enterotypes (9), whereas another study on plateau pika samples from five habitats in summer suggested that they possessed two enterotypes (4). Furthermore, studies on the gut bacteria of plateau pikas indicated that both temporal and spatial variability strongly influenced the composition, diversity, and function of the bacterial communities (28, 29). Therefore, temporal and spatial variables should be considered simultaneously to identify the enterotype categories more accurately. Enterotypes are strongly linked to host diet and physiological status (10, 14, 33). Previous studies have suggested that plateau pikas inhabiting different habitats display distinct physiological traits, especially on body mass, temperature, and resting metabolic rate (RMR) (35, 39). Additionally, food resources also shift greatly between warm and cold seasons (28). However, whether pikas with distinct physiological statuses and dietary patterns are linked to specific enterotypes is still poorly understood. Knowledge of the adaptive strategies of pikas with different enterotypes in specific habitats is also limited.

In this study, we conducted a large-scale experiment over 2 years to accurately identify the enterotype of plateau pikas. A total of 308 plateau pikas were captured from 10 altitudes during the warm (August) and cold (January) seasons. The cecal microbiota of plateau pikas were proﬁled using 16S rRNA and ITS2 sequencing. The alpha diversity of the community, relative abundance of major microbial taxa, co-occurrence networks, functional pathways, community assembly processes, and host energy metabolism in different enterotypes were compared. Additionally, shifts in host body mass, body temperature, and RMR of specific enterotypes were compared across seasons and altitudes. We aimed to determine (1) how many bacterial enterotypes can be identified in plateau pikas, and whether gut fungi can be partitioned into enterotypes, as well as differences in community composition, diversities, functions, co-occurrence networks, and assembly processes between enterotypes (2); does the proportion of different bacterial and fungal enterotypes vary with seasonal and altitude changes? (3) Do plateau pikas with different bacterial and fungal enterotypes exhibit distinct body temperatures and RMR? Do individuals with different enterotypes display distinct energy metabolism strategy to acclimatize to cold habitats?

## RESULTS

**Fig 1:**
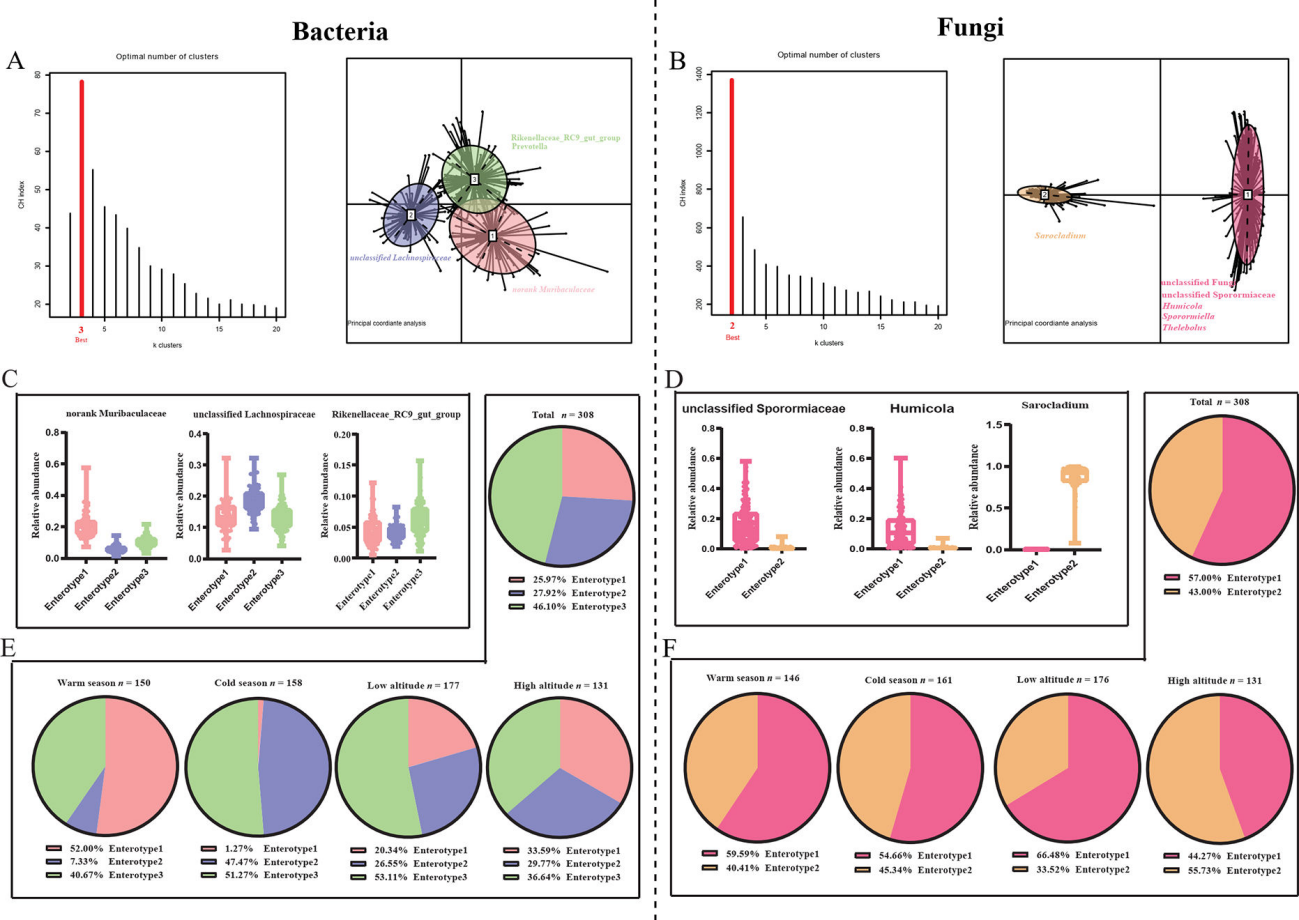
Clustering of gut microbial taxa into enterotypes and characteristics of enterotypes associated with seasonal and altitudinal distribution. (**A and B**) Enterotypes identified using Jensen–Shannon distance and partition around medoids (PAM). The left panel shows that the optimal clustering was evaluated using the CH index and the PAM method. The right panel shows the PCoA based on Jensen–Shannon distance of the microbial taxa at genus level. (**C and D**) The relative abundance of top genera in each enterotype. (**E and F**) Seasonal and altitudinal distribution of various enterotypes.

We classified 308 gut microbial samples from plateau pikas at the genus level based on the CH index. The bacterial community samples were sorted into three enterotypes, and the fungal samples were sorted into two robust enterotypes with optimal clustering (Fig. 1A and B). Additionally, the k-means clustering algorithm based on Jensen–Shannon distance also showed that the bacterial communities were sorted into three and the fungal communities were sorted into two robust groups, which used total within-cluster sum of squares (WSS) as an indicator for best clustering (Fig. S1A and B). Principal coordinate analyses (PCoA) and PCA based on the Jensen–Shannon distance at the genus level showed that the different enterotypes separated distinctly for both bacterial and fungal samples (Fig. 1A and B; Fig. S1C and D). For bacterial samples, enterotypes 1 and 2 were distinguished by norank *Muribaculaceae* and unclassified *Lachnospiraceae*, respectively, while enterotype 3 was distinguished by *Rikenellaceae_RC9_gut_group* and *Prevotella* (Fig. 1A and C). For fungal samples, enterotype 1 was distinguished by unclassified *Sporormiaceae*, *Humicola*, unclassified *Fungi*, *Sporormiella,* and *Thelebolus*, whereas enterotype 2 was distinguished by *Sarocladium* (Fig. 1B and D). Nearly half of the total bacterial samples were clustered into enterotype 3 (46.10%), while the proportion of the samples assigned to enterotypes 1 and 2 was very close, at 25.97% and 27.92%, respectively (Fig. 1E). A slightly higher proportion of total fungal samples were assigned to enterotype 1 (57.00%) compared to that of enterotype 2 (43.00%; Fig. 1F). The proportion of each enterotype varied significantly between samples from the warm and cold seasons as well as at high and low altitudes (Fig. 1E and F). For the bacterial enterotype, enterotype 1 occupied over half of the samples in the warm season (52.00%), whereas it nearly disappeared in the cold season (1.27%; Fig. 1E). The opposite trend was found in enterotype 2, which exhibited a low proportion in the warm season samples (7.33%) but increased to 47.47% in the cold season samples (Fig. 1E). Enterotype 3 was highly prevalent in the two seasons (40.67% and 51.27% for the warm and cold seasons, respectively; Fig. 1E). The importance of each enterotype also varied between samples collected at low and high altitudes (Fig. 1E). Enterotype 3 (53.11%) dominated samples at low altitudes, while enterotypes 1 (20.34%) and 2 (26.55%) were equally important (Fig. 1E). The proportions of the three enterotypes were very similar in samples from high altitudes (33.59%, 29.77%, and 36.64% for enterotypes 1, 2, and 3, respectively; Fig. 1E). Although the importance of fungal enterotypes varied between warm and cold seasons, the proportion of enterotype 1 was higher than that of enterotype 2 in samples from both warm and cold seasons (Fig. 1F). Over half of the samples at low altitudes were sorted into enterotype 1 (66.48%), and it was inverted at high altitudes, as over half of the samples were assigned to enterotype 2 (55.73%; Fig. 1F). Additionally, we found that the ratio of enterotypes for both bacterial and fungal samples did not differ significantly between females and males, except for samples from the warm season, which showed that bacterial enterotype 1 occurred at a higher proportion in male individuals than other enterotypes (Fig. S2). For the fungal samples, we detected that a higher proportion of females displayed enterotype 1 than enterotype 2 profiles (Fig. S2).

To determine the interaction between bacterial and fungal taxa, we analyzed the correlation between bacterial and fungal taxa at genus levels based on Spearman’s correlation (Fig. S3). However, we found that the interactions between bacterial and fungal taxa in our samples were weaker compared with the interactions between bacterial taxa or fungal taxa themselves (|*r*| > 0.6, *P* < 0.05; Fig. S3). Although the two bacterial genera, norank Erysipelotrichaceae and unclassified Erysipelotrichaceae, displayed strongly correlations with many fungal genera, they showed a very low relative abundance in all samples (<0.01; Fig. S3C; Table S1). Additionally, the relative abundance of these bacterial genera that displayed a significant correlation with fungal taxa was lower than 0.01 (Table S1). In addition, to determine the correlation between bacterial and fungal enterotypes, we analyzed the proportion of fungal enterotypes in each bacterial enterotype (Fig. S4). In total, most of the pikas with fungal enterotype 2 possessed bacterial enterotypes 1 and 2, while individuals with fungal enterotype 1 possessed bacterial enterotype 3 (Fig. S4A). The similar results also found in the samples from different seasons and elevations (Fig. S4B, C, D, and E), suggesting that this correlation did not change with the variation of environment.

**Fig 2:**
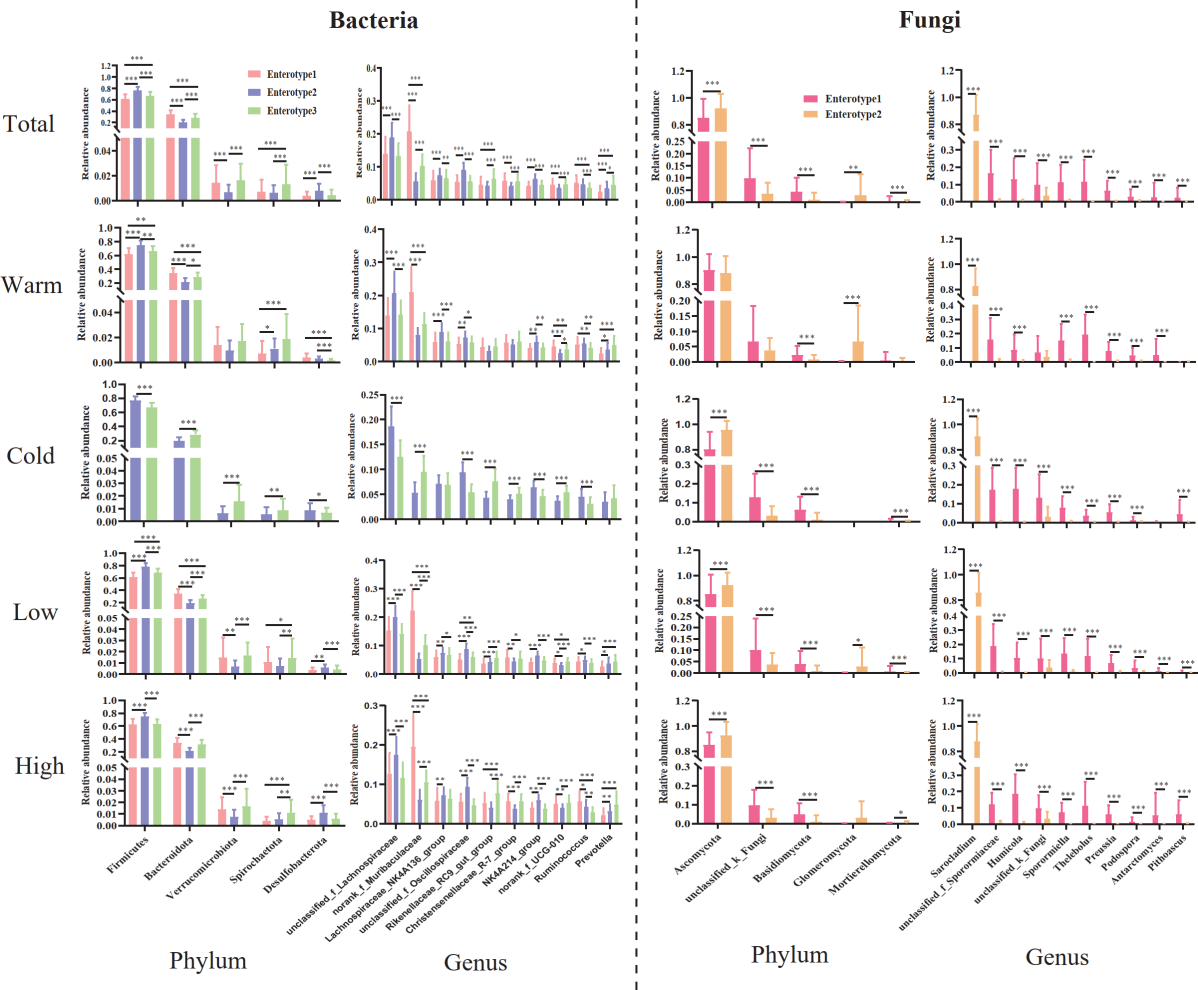
The variation of dominant microbial phyla and genera between enterotypes. Comparison of the difference in relative abundance of major phyla and genera between enterotypes for bacterial and fungal communities from total, warm and cold season, and low- and high-altitude samples, respectively. The asterisks indicate *P < 0.05, **P < 0.01, ***P < 0.001 (Mann-Whitney *U* and Kruskal-Wallis tests).

For total samples, *Firmicutes* and *Bacteroidetes* were the top two abundant bacterial phyla in all enterotypes, and pikas with enterotype 2 harbored the highest abundance of *Firmicutes* (Kruskal-Wallis test, *χ* ^2^ = 129.823, *P <* 0.001), while *Bacteroidetes* was more abundant in pikas with enterotype 1 (Kruskal-Wallis test, *χ* ^2^ = 135.755, *P <* 0.001; Fig. 2). This advantage did not change with shifts in seasons and altitudes (Fig. 2). *Ascomycota*, which was more abundant in pikas with enterotype 2 than enterotype 1 in all groups (Mann-Whitney *U* test, *Z* = −6.951, *P <* 0.001) except for the warm season group, dominated the two fungal enterotypes in all groups (Fig. 2). Norank *Muribaculaceae* was the top abundant genus in enterotype 1 and was significantly higher than in enterotypes 2 and 3 (Kruskal-Wallis test, *χ* ^2^ = 204.899, *P <* 0.001; Fig. 2). Unclassified *Lachnospiraceae* was the top abundant bacteria in enterotype 2 and was significantly higher than that of the other two enterotypes (Kruskal-Wallis test, *χ* ^2^ = 78.199, *P <* 0.001; Fig. 2). *Rikenellaceae_RC9_gut_group* (Kruskal-Wallis test, *χ* ^2^ = 50.379, *P <* 0.001) and *Prevotella* (Kruskal-Wallis test, *χ* ^2^ = 43.865, *P <* 0.001) were abundant in enterotype 3 and significantly higher than the other enterotypes (Fig. 2). Similarity patterns were detected in all sub-groups (samples from warm and cold seasons and low and high altitudes, respectively; Fig. 2). For total fungal samples, *Sarocladium* predominated the fungal taxa in pikas with enterotype 2 and was significantly higher than that of enterotype 1 (Mann-Whitney *U* test, *Z* = −16.07, *P <* 0.001; Fig. 2), whereas the dominant fungal taxa of enterotype 1 mainly belonged to several genera, including unclassified Sporormiaceae (Mann-Whitney *U* test, *Z* = −13.914, *P <* 0.001), *Humicola* (Mann-Whitney *U* test, *Z* = −14.02, *P <* 0.001), and *Sporormiella* (Mann-Whitney *U* test, *Z* = −14.266, *P <* 0.001), which was significantly higher than that of enterotype 2 (Fig. 2). Similar results can also be found in all sub-groups (Fig. 2).

**Fig 3:**
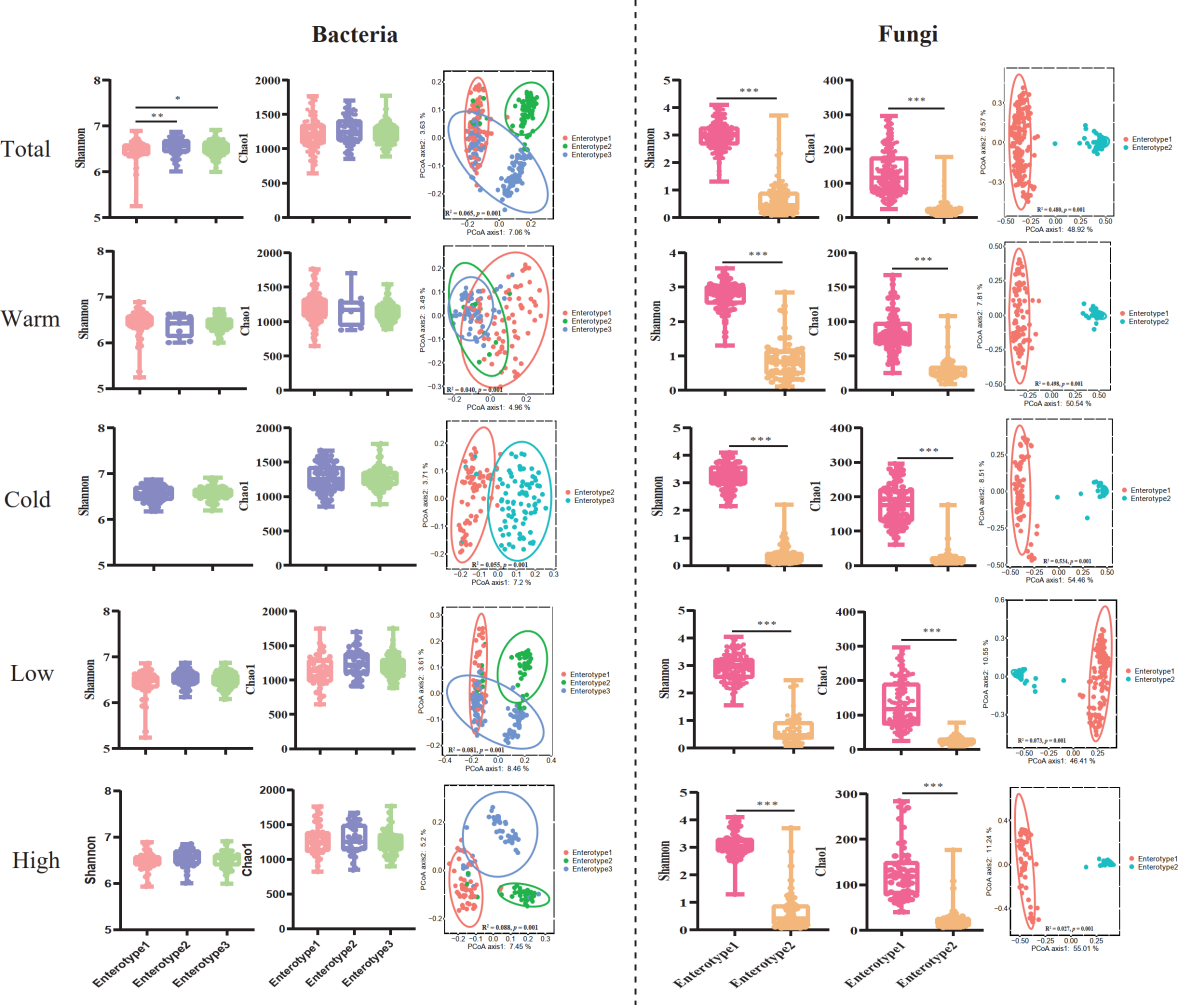
The variation in community diversities between enterotypes. Comparison indicating the Shannon and Chao 1 indices between enterotypes and PCoA based on Bray–Curtis distance at amplicon sequence variant (ASV) level for bacterial and fungal communities from total, warm and cold season, and low and high altitude samples, respectively. The asterisks indicate *P < 0.05, **P < 0.01, ***P < 0.001 (one-way ANOVA, Mann-Whitney *U,* and Kruskal-Wallis tests).

The alpha diversity of the gut microbiota varied significantly between pikas with different enterotypes (Fig. 3). For the total bacterial samples, pikas with enterotypes 2 and 3 harbored a higher Shannon index than those with enterotype 1 (one-way ANOVA test; *F*_2, 307_ = 4.371, *P* = 0.013), while the Chao 1 index did not differ between enterotypes (Kruskal-Wallis test, *χ* ^2^ = 2.878, *P* = 0.237; Fig. 3). For the total fungal samples, pikas belonging to enterotype 1 harbored higher Shannon (Mann-Whitney *U* test, *Z* = −14.395, *P* = 0.000) and Chao 1 (Mann-Whitney *U* test, *Z* = −14.231, *P <* 0.001) indices than that of enterotype 2 (Fig. 3). The PCoA based on Bray-Curits distance showed that the taxa belonging to different enterotypes were significantly separated for both the bacterial (PERMANOVA, *R* ^2^ = 0.065, *P* = 0.001) and fungal (PERMANOVA, *R* ^2^ = 0.480, *P* = 0.001) communities (Fig. 3). However, we did not find a significant difference in the alpha diversity between enterotypes for the bacterial samples from warm (one-way ANOVA test, *F*_2, 307_ = 0.471, *P* = 0.625; Kruskal-Wallis test, Chao 1, *χ* ^2^ = 3.301, *P* = 0.220) and cold (Mann-Whitney *U* test, Shannon, *Z* = −0.125, *P* = 0.900; Chao 1, *Z* = −0.147, *P* = 0.883) seasons, and low (Kruskal-Wallis test, Shannon, *χ* ^2^ = 3.926, *P* = 0.140; Chao 1, *χ* ^2^ = 4.871, *P* = 0.088) and high (one-way ANOVA test, Shannon, *F*_2, 307_ = 0.873, *P* = 0.610; Chao 1, *F*_2, 307_ = 0.227, *P* = 0.797) altitudes (Fig. 3), but the beta diversity differed significantly between enterotypes in all sub-groups (Fig. 3). For the fungal community, the Shannon and Chao 1 indices displayed consistent patterns in all sub-groups compared with that of total samples (Fig. 3). Additionally, beta diversity was markedly different between enterotypes in all subgroups (Fig. 3).

**Fig 4:**
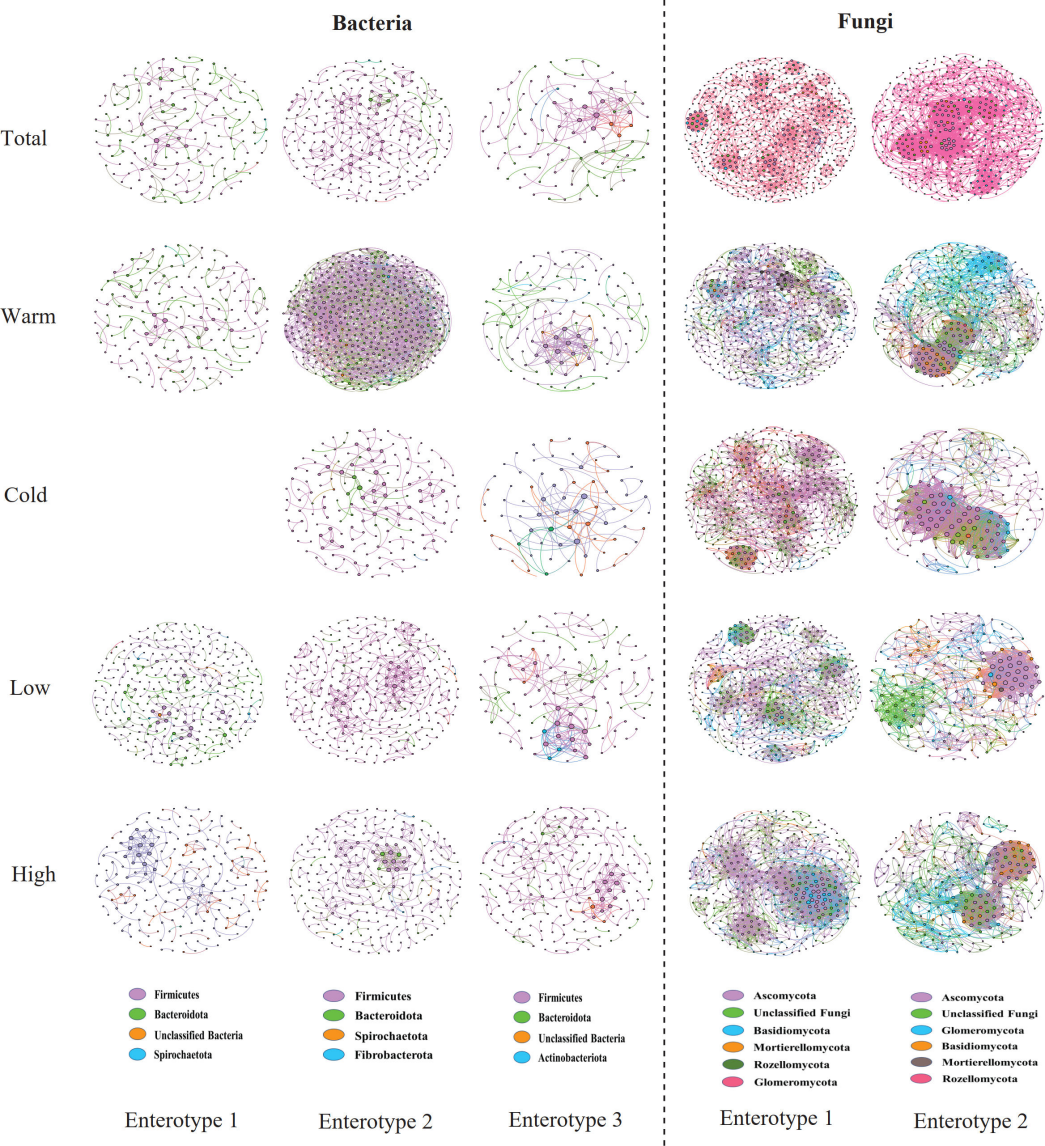
Co-network of different enterotypes in plateau pikas based on Spearman’s correlation analysis between ASVs. Each connection displays a correlation coefficient >|0.7| for bacterial taxa and |0.5| for fungal taxa, and a *P* value < 0.05. The size of each node is proportional to the number of connections. The nodes were colored by taxonomy at phylum levels.

The Spearman correlation-based co-occurrence network analysis showed that pikas with enterotype 2 harbored more nodes and edges in total samples and all sub-groups, suggesting that enterotype 2 possessed more complex bacterial networks than enterotypes 1 and 3 (Fig. 4; Table S2). Most taxa in the networks belonged to Firmicutes and Bacteroidetes. The proportion of taxa belonging to Firmicutes was higher than that of Bacteroidetes. Additionally, nearly all of the links in the bacterial networks were positively correlated in all groups, except for individuals with enterotype 2 in the warm season group, which showed a high proportion of negative correlation between taxa (30.69%; Fig. 4; Table S2). Interestingly, we found that pikas with enterotype 1 possessed higher modularity than the other two enterotypes in the total group and all sub-groups (Fig. 4; Table S2). For the fungal networks, individuals with enterotype 1 displayed more nodes than those with enterotype 2, while enterotype 2 had more edges than those with enterotype 1, except for the samples from the cold season and high altitude. We only detected positive links between the taxa in the fungal networks of enterotype 2, and a few negative links were detected in enterotype 1. The modularity of networks of enterotype 1 was higher than that of enterotype 2 in the total samples and all sub-groups. However, enterotype 2 in the high-altitude group showed higher modularity than that of enterotype 1 (Fig. 4; Table S3). Most nodes in the fungal networks belonged to the phyla Ascomycota, Basidiomycota, and unclassified Fungi, while Glomeromycota played an important role in the network nodes of enterotype 2 (Fig. 4; Table S3).

**Fig 5:**
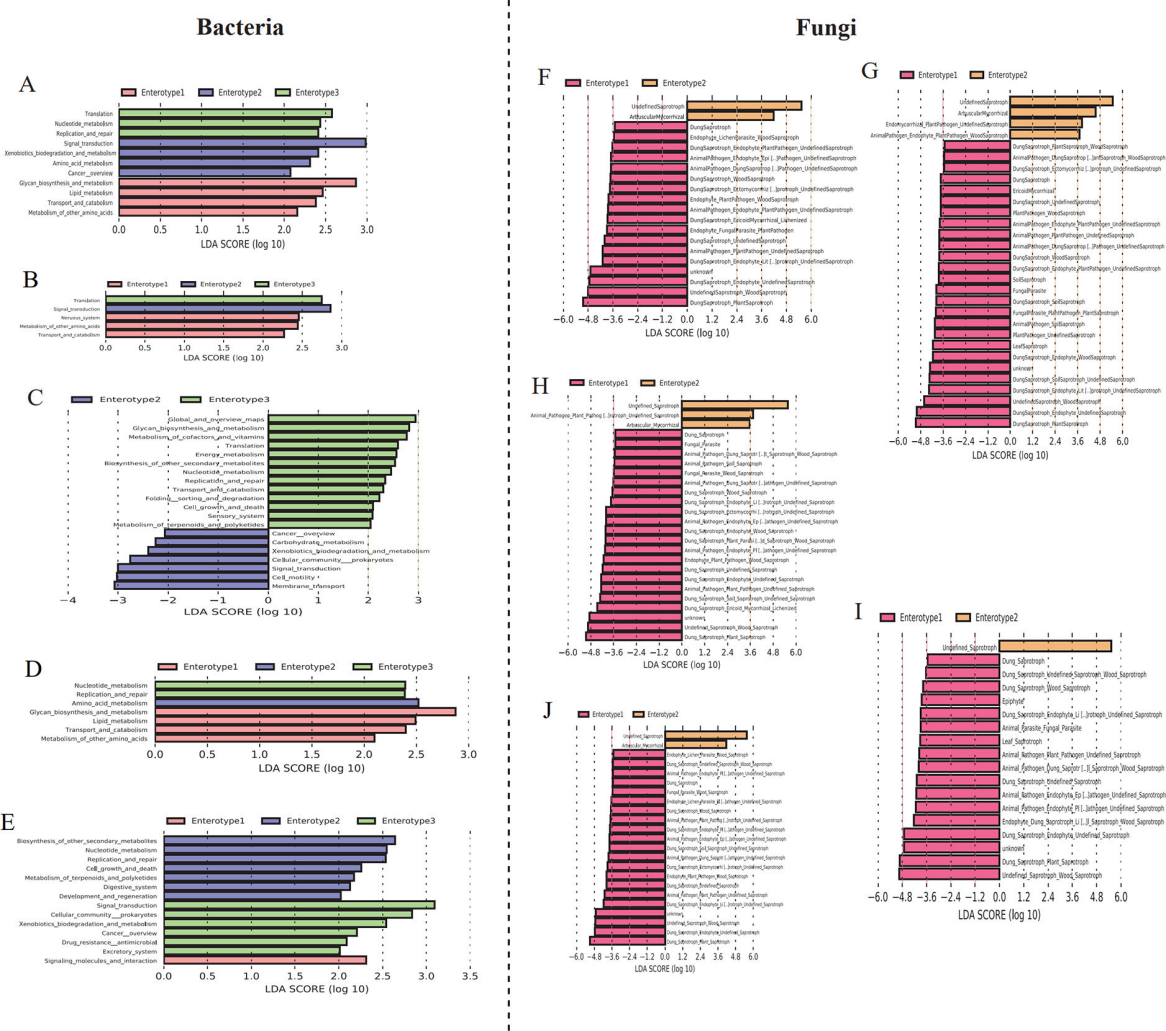
Functional prediction of the microbial community from different enterotypes. Bar chart showing the log-transformed LDA scores of functional pathways identified using LEfSe analysis as associated with each pika enterotype. The LEfSe analysis for the total samples (**A and F**), the samples from warm (**B and G**) and cold (**C and H**) seasons, and low (**D and I**) and high (**E and J**) altitudes.

To detect differences in the functional capacity of different enterotypes, we performed PICRUSt2 analysis using Kyoto Encyclopedia of Genes and Genomes (KEGG) and FUNGuild databases for bacterial and fungal communities, respectively. In the bacterial community, pathways related to nutrition metabolism, such as lipid metabolism, glycan biosynthesis and metabolism, and metabolism of other amino acids, were enriched in pikas with enterotype 1, while the pathways including amino acid metabolism and xenobiotic biodegradation and metabolism were enriched in enterotype 2 (Fig. 5A). However, nucleotide metabolism and the two pathways associated with genetic information processing were enriched in enterotype 3 (Fig. 5A). The differences in functional capacity varied among the different sub-groups. In the summer samples, the functional differences between enterotypes were minor, and only one pathway, metabolism of other amino acids, was associated with nutritional metabolism enriched in enterotype 1 (Fig. 5B). In contrast, 20 pathways differed between enterotypes in the cold season samples (Fig. 5C). Seven functional pathways, including carbohydrate metabolism and xenobiotic biodegradation and metabolism, were enriched in enterotype 2, while 13 pathways, such as glycan biosynthesis and metabolism and energy metabolism, were enriched in enterotype 3 (Fig. 5C). For the samples at low altitude, amino acid metabolism was enriched in enterotype 2, while lipid metabolism and metabolism of other amino acids were more abundant in enterotype 1 (Fig. 5D). Although the difference in functional capacity between enterotypes at high altitudes was larger than that at low altitudes, we did not detect a difference in the pathways related to nutritional metabolism between enterotypes (Fig. 5E). Additionally, the results of the Kruskal-Wallis test showed that the top 10 abundant pathways differed significantly between enterotypes (Fig. S5). For example, pikas with enterotype 2 harbored more pathways, including carbohydrate metabolism (Kruskal-Wallis test, *χ* ^2^ = 24.687, *P <* 0.001) and amino acid metabolism (Kruskal-Wallis test, *χ* ^2^ = 79.813, *P <* 0.001), than the other two enterotypes, whereas pikas with enterotype 3 possessed more pathways, including energy metabolism (Kruskal-Wallis test, *χ* ^2^ = 27.149, *P <* 0.001) and glycan biosynthesis and metabolism (Kruskal-Wallis test, *χ* ^2^ = 96.289, *P <* 0.001) than enterotype 2 (Fig. S5).

Differences in fungal functions between enterotypes were compared using the FUNGuild database. LEfSe analysis showed that many fungal functions in enterotype 1 were more abundant than that in enterotype 2, while only a few fungal functions were enriched in enterotype 2 (Fig. 5F, G, H, I and J). Similar results were observed in all sub-groups. Additionally, the trophic mode of most of the enriched fungal taxa in enterotype 1 belongs to dung saprotrophs and animal pathogens (Fig. 5F, G, H, I, and J).

**Fig 6:**
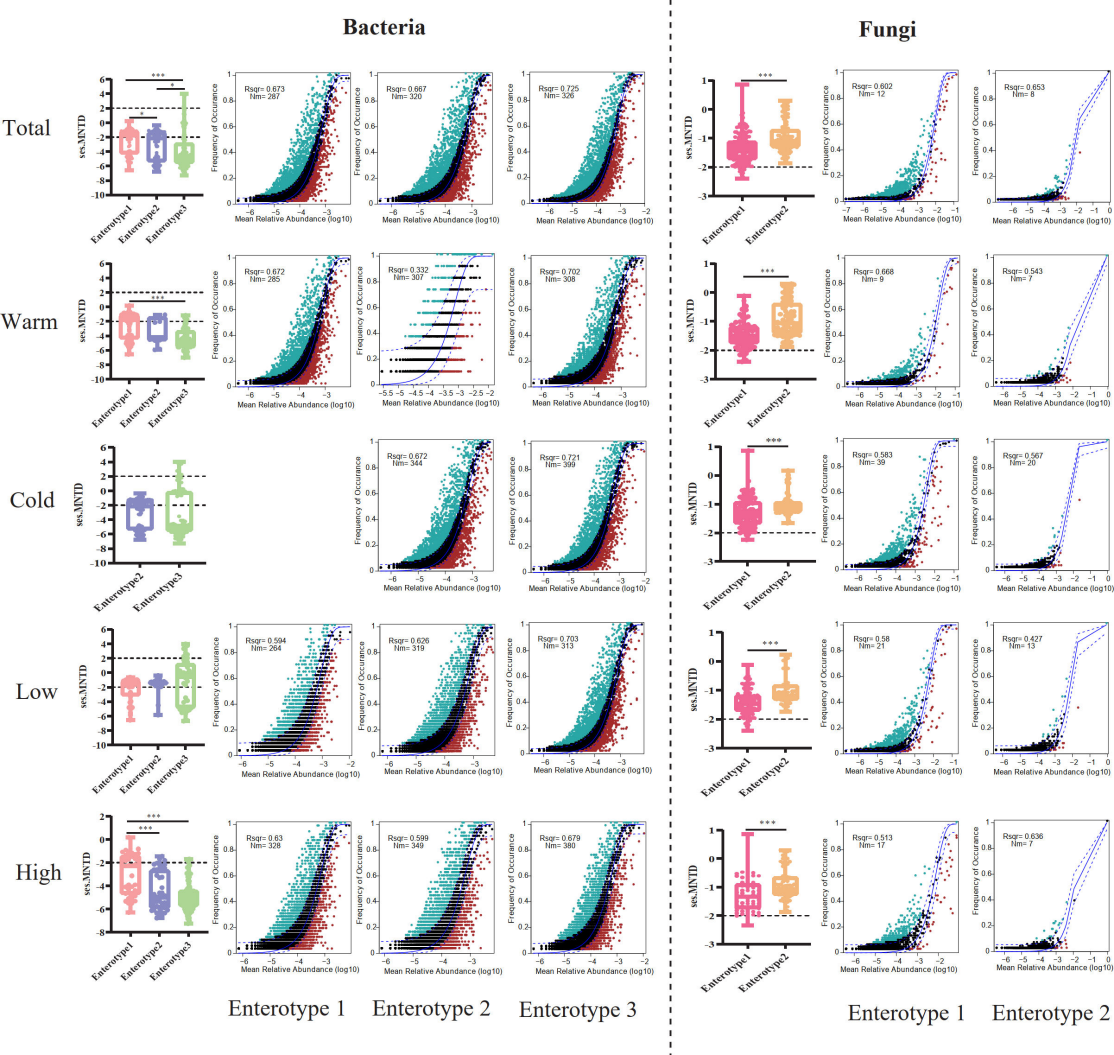
The analysis of community assembly processes for enterotypes. The first panel on the left shows that ses.MNTD was used to determine the assembly processes of microbial communities in a pika. The remaining three panels on the right show that the neutral model was applied to test the importance of stochastic processes instructing community assembly between metacommunities. The analysis was conducted for total, warm and cold season, and low- and high-altitude samples. The asterisks indicate * *P* < 0.05, **P < 0.01, ***P < 0.001 (Mann-Whitney *U* and Kruskal-Wallis tests).

For total bacterial samples, over half of the ses.MNTD values (56.25%) in enterotype 1 were between −2 and 2, indicating that the gut bacterial taxa in these plateau pikas were randomly distributed, while 43.75% of the values were lower than −2, suggesting that the bacterial taxa in these individuals were phylogenetically clustered (Fig. 6). The results were inverted in enterotype 2, which showed that over half of the values (53.49%) were lower than −2 and 46.51% of the values were between −2 and 2 (Fig. 6). Most values (78.17%) in enterotype 3 were lower than −2, implying a greater degree of phylogenetic clustering of bacterial taxa in these individuals (Fig. 6). We also observed that a small proportion of the ses.MNTD values (5.63%) in enterotype 3 was over 2, suggesting that the bacterial taxa in these pikas were phylogenetically over dispersed (Fig. 6). Additionally, the gut bacterial communities in the three enterotypes showed a good fit to the neutral community model (Fig. 6). Neutral processes explained 67.3%, 66.7%, and 72.5% of the community variation for enterotypes 1, 2, and 3, respectively, in the total samples, suggesting that neutral or stochastic processes were more important in enterotype 3 than in the other two enterotypes (Fig. 6). Similar results were also found in all sub-groups, except for the ses.MNTD values for the samples at low altitude and the neutral processes for the samples in the warm season (Fig. 6). Nearly all ses.MNTD values (93.62%) in enterotype 2 from low altitude samples were between −2 and 2 (Fig. 6), implying a greater degree of random distribution of bacterial taxa, which opposed the results obtained from total and other sub-group samples. The neutral processes only explained 33.2% of the community variation in enterotype 2 from the warm season samples (Fig. 6), indicating that deterministic processes were over stochastic processes in structuring community assembly.

In contrast with the bacterial community, most ses.MNTD values in all fungal groups were between −2 and 2, suggesting that fungal taxa were mainly randomly distributed in the gut ecosystem of plateau pikas (Fig. 6). We detected only a small proportion of ses.MNTD values (6.29%) in enterotype 1 was less than −2 and all ses.MNTD values in enterotype 2 from total samples were between −2 and 2, indicating that stochastic processes overwhelm deterministic processes in governing the fungal community assembly processes. Similar results were also detected in all sub-groups (Fig. 6). The neutral model was well-fitted for the fungal communities of the two enterotypes in all groups (Fig. 6). Overall, neutral processes were more important in two enterotypes (enterotype 1: 60.2%, enterotype 2: 65.3%) from total samples, enterotype 1 (66.8%) from warm season samples, and enterotype 2 (63.6%) from high altitude samples, than that of others, while neutral processes only explained 42.7% of the community variation for enterotype 2 at low altitudes (Fig. 6).

**Fig 7:**
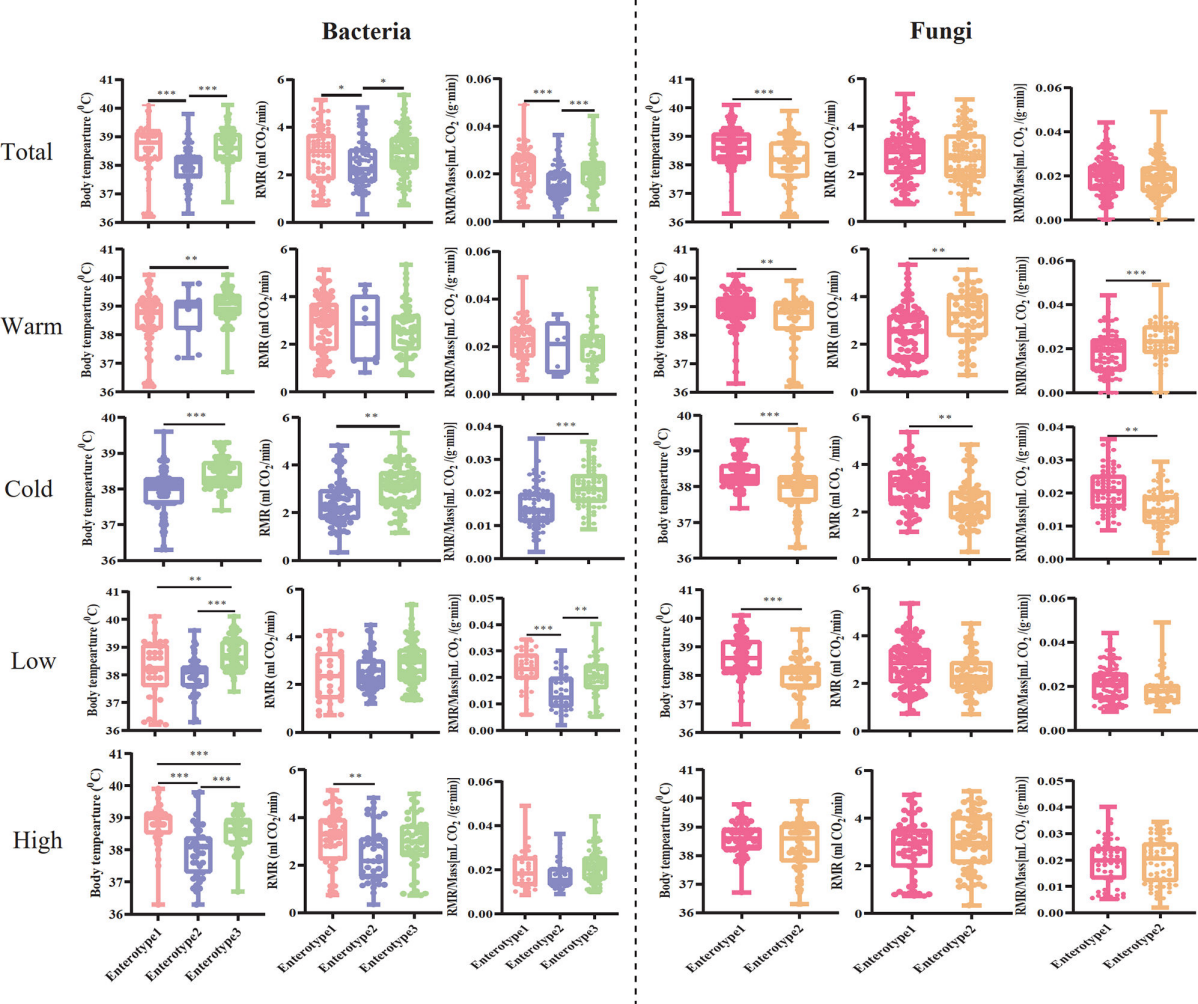
Comparison host-related physiological features between enterotypes. Host body temperature, RMR, and per unit mass of RMR (RMR/Mass) were compared between enterotypes for total, warm and cold season, and low- and high-altitude samples. The asterisks indicate *P < 0.05, **P < 0.01, ***P < 0.001 (Mann-Whitney *U* and Kruskal-Wallis tests).

We compared the differences in host body temperature, RMR, and RMR/Mass (per unit mass of RMR) between enterotypes to determine whether plateau pikas with different enterotypes displayed a distinct physiological status. The results showed that all three parameters measured in pikas with bacterial enterotypes 1 and 3 were higher than those with enterotype 2 (Kruskal-Wallis test, *χ* ^2^ = 53.273, *P <* 0.001; RMR, *χ* ^2^ = 6.372, *P* = 0.041; RMR/Mass, *χ* ^2^ = 22.867, *P <* 0.001; Fig. 7). However, the association between enterotypes and host physiological status varied among samples from different seasons and altitudes (Fig. 7). For example, the RMR did not differ between enterotypes in the warm season samples (Kruskal-Wallis test, *χ* ^2^ = 1.974, *P* = 0.373), and pikas with enterotype 3 harbored significantly higher body temperatures than those with enterotype 1 (Kruskal-Wallis test, *χ* ^2^ = 10.944, *P* = 0.004; Fig. 7). For the fungal samples, we found that pikas with enterotype 1 had higher body temperatures than those with enterotype 2 (Mann-Whitney *U* test, *Z* = −5.323, *P <* 0.001), except for pikas from high altitudes, which did not show differences between enterotypes (Mann-Whitney *U* test, *Z* = −1.147, *P* = 0.251; Fig. 7). Pikas with enterotype 2 from warm season samples exhibited higher RMR than did individuals with enterotype 1 (Mann-Whitney *U* test, *Z* = −3.438, *P* = 0.001), while the situation appeared inverse for pikas during the cold season (Mann-Whitney *U* test, *Z* = −2.858, *P* = 0.004; Fig. 7). Interestingly, we found that host body mass also significantly differed between enterotypes (Fig. S6). Overall, pikas with bacterial enterotype 2 usually displayed the greatest body mass compared to those with enterotypes 1 and 3 (Kruskal-Wallis test, *χ* ^2^ = 27.350, *P <* 0.001). However, pikas with both enterotypes 2 and 3 exhibited greater body mass than those with enterotype 1 from low altitudes (Kruskal-Wallis test, *χ* ^2^ = 9.989, *P* = 0.007), while we did not detect differences between enterotypes in samples obtained during the warm season (Kruskal-Wallis test, *χ* ^2^ = 2.672, *P* = 0.263; Fig. S6). For the fungal samples, pikas with enterotype 2 showed greater body mass than those with enterotype 1 (Mann-Whitney *U* test, *Z* = −3.194, *P* = 0.001), except for the samples from low altitudes, which displayed non-significant differences between enterotype 1 and enterotype 2 (Mann-Whitney *U* test, *Z* = −0.541, *P* = 0.589; Fig. S6).

**Fig 8:**
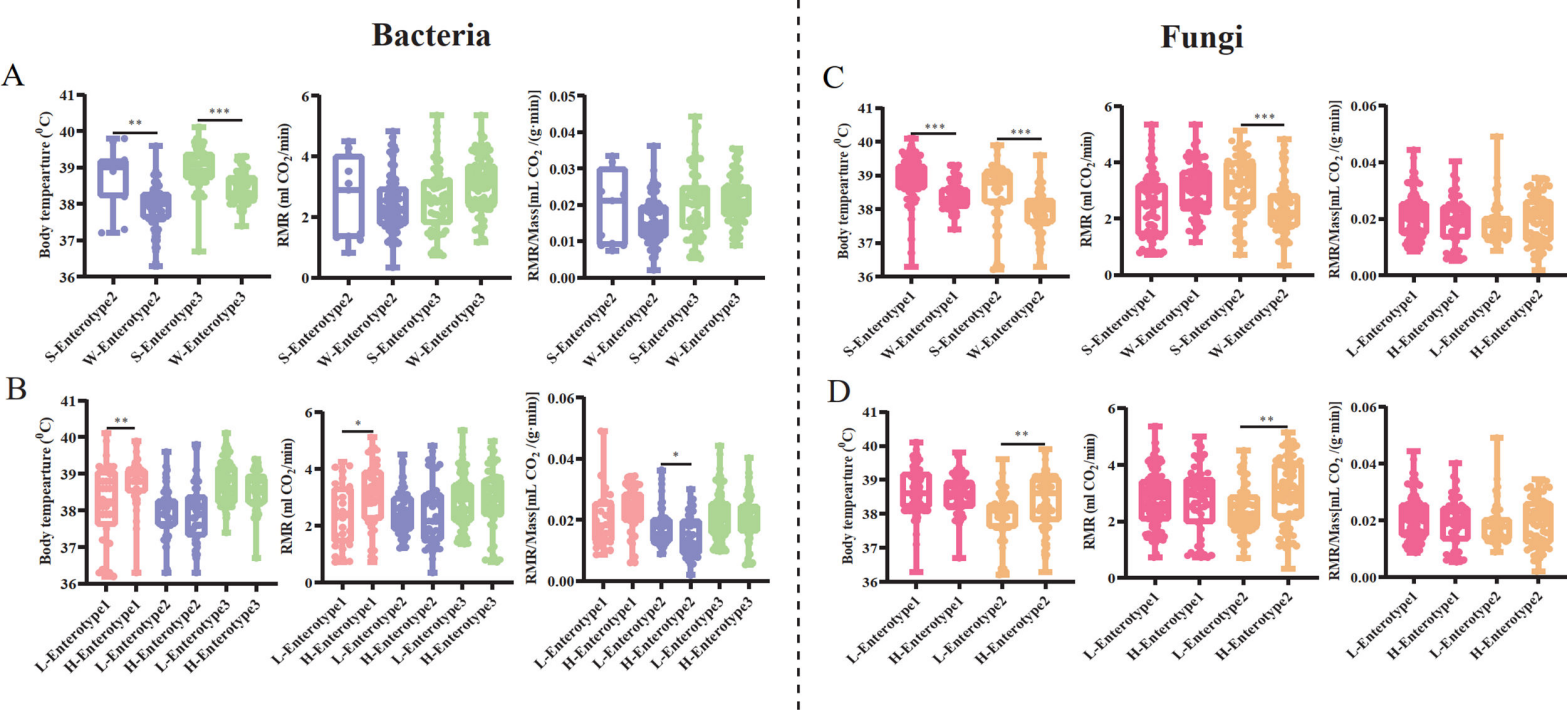
The effect of seasons and altitudes on host-related physiological features within each enterotype. Comparisons between host body temperature, RMR, and RMR/Mass between warm and cold seasons (**A and C**) and low and high altitudes (**B and D**) within each enterotype. The asterisks indicate * *P* < 0.05, **P < 0.01, ***P < 0.001 (Mann-Whitney *U* and Kruskal-Wallis tests).

The host body temperature, RMR, and RMR/Mass of pikas within each enterotype also varied significantly across seasons and altitudes (Fig. 8). The body temperatures of pikas with bacterial enterotypes 2 (Mann-Whitney *U* test, *Z* = −3.059, *P* = 0.002) and 3 (Mann-Whitney *U* test, *Z* = −6.828, *P <* 0.001) were lower in the cold season than in the warm season (Fig. 8A). Of the three bacterial enterotypes, we detected that the host body temperature (Mann-Whitney *U* test, *Z* = −2.660, *P* = 0.008) and RMR (Mann-Whitney *U* test, *Z* = −2.570, *P* = 0.010) of pikas with enterotype 1 significantly increased at high altitudes (Fig. 8B), whereas RMR/Mass significantly decreased in pikas with enterotype 2 at high altitudes (Mann-Whitney *U* test, *Z* = −2.511, *P* = 0.012; Fig. 8B). For the fungal enterotypes, the physiological status of pikas with enterotype 1 was relatively stable between seasons since we only detected that host body temperatures significantly decreased in the cold season compared to the warm season (Mann-Whitney *U* test, *Z* = −6.715, *P <* 0.001; Fig. 8C). The body temperature (Mann-Whitney *U* test, *Z* = −4.848, *P <* 0.001), RMR (Mann-Whitney *U* test, *Z* = −3.875, *P <* 0.001), and RMR/Mass (Mann-Whitney *U* test, *Z* = −5.545, *P <* 0.001) of pikas with enterotype 2 all significantly decreased in the cold season compared to the warm season (Fig. 8C), while these parameters increased at high altitudes (Mann-Whitney *U* test, body temperature, *Z* = −3.119, *P* = 0.002; RMR, *Z* = −2.826, *P* = 0.005; Fig. 8D), except for RMR/Mass (Mann-Whitney *U* test, *Z* = −1.013, *P* = 0.311; Fig. 8D). Additionally, we detected a significant increase in host body mass in the cold season for both bacterial and fungal enterotypes (Fig. S7A and C). Pikas with bacterial enterotype 1 (Mann-Whitney *U* test, *Z* = −2.231, *P* = 0.026), enterotype 2 (Mann-Whitney *U* test, *Z* = −4.646, *P <* 0.001), and fungal enterotype 2 showed greater body masses at high altitudes (Mann-Whitney *U* test, *Z* = −3.987, *P <* 0.001; Fig. S7B and D).

## DISCUSSION

We identified three enterotypes from the bacterial community and two enterotypes from the fungal community in the gut of plateau pikas (Fig. 1A and B). Studies on human beings (8), chimpanzees (11), African buffalo (*Syncerus caffer*) (13), pigs (12), and honeybees (15) have also identified enterotypes from the gut microbiota of these animals, indicating that enterotypes are ubiquitous in human and animal gut ecosystems. In addition, enterotypes are highly species identifiable. For example, three human enterotypes were identified as *Bacteroides*, *Prevotella*, and *Ruminococcus*, respectively (8), while *Lactobacillus*, *Prevotella Sarcina*, and *Turicibacter Clostridium sensu stricto* were the three enterotype genera identified in pigs (12). Interestingly, two previous studies on enterotypes of plateau pikas showed inconsistent results; one suggested that plateau pikas harbored three enterotypes (9), while another showed that they only had two enterotypes (4). The inconsistent results were probably attributed to the sample size of the two studies based on seasonal and geographic shifts. These inconsistent results can also be found in studies regarding the human gut microbiome. For example, research on adults from six nationalities in European countries identified three enterotypes (8), while sample collection from humans from seven countries on six continents, with an age range from infant to adult, identified four enterotypes (34). In this study, we considered both seasonal and geographic variables during sample collection. We collected 308 samples from 10 sample sites during two seasons. Additionally, research on the oral microbiome of adolescents identified two “stomatotypes” based on the protocol of enterotype clustering (16), which suggested that the method could also be used to analyze bacterial communities from different ecosystems or taxa. Indeed, we identified two enterotypes from the fungal community in the gut of plateau pikas (Fig. 1B). Similar results were also found in studies on human beings, which showed that gut mycobiomes separated significantly between residents according to geography and ethnicity (40).

The results showed that the proportions of different enterotypes were distinct (Fig. 1E). Different enterotypes are usually closely associated with different dietary patterns (10). The norank *Muribaculaceae*-dominant enterotype (enterotype 1) displayed the highest abundance of norank *Muribaculaceae* (Fig. 2). A study on mice showed that a high-fat diet can significantly increase the abundance of norank *Muribaculaceae* (41), indicating that enterotype 1 was strongly associated with a long-term high-fat diet. Function prediction confirmed this speculation, as the lipid metabolism pathway was significantly enriched in enterotype 1. Unclassified *Lachnospiraceae* is a well-known butyrate-producing family dominating enterotype 2 (42) (Fig. 2). Additionally, we observed a higher abundance of unclassified *Oscillospiraceae*, which is closely associated with acetate production (43), in enterotype 2 compared with enterotypes 1 and 3 (Fig. 2). Enterotype 3 was enriched with bacterial genera, including *Rikenellaceae_RC9_gut_group* and *Prevotella*, compared to the other two enterotypes (Fig. 2). The *Rikenellaceae_RC9_gut_group* is known to produce short-chain fatty acids, such as propionate and acetate, which may provide energy for the host or as a signal molecule regulating host energy metabolism (44). Previous studies on the human gut microbiome have suggested that the *Prevotella*-dominated enterotype is closely associated with long-term carbohydrate dietary patterns (10). Additionally, the results of function prediction showed that pathways including carbohydrate metabolism and energy metabolism were enriched in enterotypes 2 and 3, respectively, which confirmed that pikas with the two enterotypes probably engaged in a high-fiber diet. For the fungal taxa, certain genera predominated in the two enterotypes, including *Sarocladium*, unclassified *Sporormiaceae*, *Humicola*, *Sporormiella*, and *Thelebolus*, and were not shared with other mammals, such as humans (26), pigs (45), and mice (40), which suggest that pikas may harbor unique gut fungal taxa for adaptation to high altitudes. Interestingly, *Sporormiella* can act as an indicator of host engagement in coprophagy (46). Plateau pikas are typical small mammals that consume not only the feces of conspecific individuals but also other species, such as yaks (35). Therefore, *Sporormiella* enriched in pikas with fungal enterotype 1 may indicate that they engage in coprophagy more often than pikas with enterotype 2.

Our results showed that gut bacterial community diversity was distinct among the three enterotypes (Fig. 3). Many studies have indicated that numerous environmental variables (e.g., diet, temperature, and rainfall) or host-related features (e.g., sex, age, reproductive state, and body temperature) significantly affect gut bacterial community diversity (5, 47 – 49). Thus, the distinct gut bacterial diversity between individuals suggests that they are exposed to different environments or have distinct physiological statuses (30, 50 – 53). Indeed, we detected higher alpha-diversity in pikas with enterotypes 2 and 3 than those with enterotype 1 (Fig. 3), which was attributed to the significantly higher proportion of the two enterotypes detected during the cold season than in the warm season. The low ambient temperature and fiber-rich diet during the cold season resulted in higher alpha-diversity of gut bacteria in wild plateau pikas than that observed during the warm season (28, 29). Interestingly, pikas with fungal enterotype 1 harbored significantly higher fungal alpha diversity than those with enterotype 2 (Fig. 3). As discussed above, pikas with enterotype 1 possibly engaged in coprophagy more frequently, which may increase their gut fungi diversity via horizontal transformation (54, 55).

Overall, the gut bacterial co-occurrence networks of pikas with enterotype 2 were more complex and stable than the other two enterotypes (Fig. 4), since the network topological parameters, including network size, connectivity, average clustering coefficient, and modularity (which indicated greater stability), were higher in enterotype 2 than in other enterotypes (56). A previous study on soil microbial networks showed that climate warming significantly increased the complexity and stability of the network (57). However, we found that pikas with enterotype 2 displayed a significantly lower body temperature than that of pikas with the other two enterotypes (Fig. 7). These inconsistent results may be attributed to the fact that both environmental variables and host-related features affect gut microbiota (58, 59). For example, results indicate that a large proportion of pikas with enterotype 2 from the cold season consumed high levels of fiber, and fiber-rich food can increase the gut bacterial network complexity and stability (51). Interestingly, pikas with fungal enterotype 1 displayed higher stability of fungal co-occurrence networks, while enterotype 2 pikas showed more complex networks (Fig. 4 and Table S3). Hosts can benefit from stable microbial networks (60).

The intestinal tracts of animals display some similarities to isolated oceanic islands and exhibit unique niches that are distinct from the external environment, which may impose high selection pressure on the microbial taxa colonized in the gut ecosystems (61, 62), resulting in high phylogenetic clustering for the coexisting taxa. However, previous studies have demonstrated that host development stage, host diet, and habitat significantly affect the phylogenetic distance of gut microbial taxa (62 – 64). Most of the ses.MNTD values for the bacterial enterotype 1 group were between −2 and 2, indicating that stochastic processes (phylogenetic random) dominate assemblages of microbial communities in pikas with enterotype 1 (65). In contrast, the results of the null models suggest that the gut bacterial taxa in enterotypes 2 and 3 showed high phylogenetic clustering (Fig. 6). A large proportion of pikas from the warm season were sorted into enterotype 1 (Fig. 1E). Food resource diversity and richness are high during the warm season (28), which can provide more food niches for different bacterial taxa, resulting in high random community assembly (63). Correspondingly, we detected a higher proportion of enterotypes 2 and 3 in the cold season when food resource diversity and richness decreased. A higher proportion of fiber in the diet during the cold season could contribute to bacterial taxa colonization and reproduction (51). Functionally similar taxa usually have short phylogenetic distances (66, 67). For the fungal taxa, although a small part of the ses.MNTD values in enterotype 1 were lower than −2, most of the values in enterotype 1 and all values in enterotype 2 were between −2 and 2, indicating that the fungal taxa in the pika gut ecosystems were dominated by stochastic processes (65). A high proportion of environmental fungal taxa, such as *Sporormiella*, can randomly colonize host gut ecosystems (54, 55), which probably contributes to the phylogenetic randomness of the gut fungal community, resulting in highly stochastic community assembly processes (65).

The Sloan neutral model has been used to investigate the importance of stochastic processes in structuring community assemblies (68). Both the bacterial and fungal communities for all enterotypes fit well to the neutral model (Fig. 6), indicating that stochastic processes play an important role in community composition turnover between pikas. For the bacterial community, the stochastic process was more important in structuring the community assembly in pikas with enterotype 3 (Fig. 6). One of the possible reasons is that a large proportion of pikas with enterotype 3 were from low-altitude habitats where pikas were more active, which could increase the chance of horizontal transfer of gut bacteria taxa between pikas by touching or consuming conspecific feces (31, 35). For fungal taxa, the stochastic process is important in governing community assembly, probably because many environmental fungal taxa can randomly transfer and colonize gut ecosystems (54, 55).

The gut microbiota plays an important role in host energy metabolism (32). Our results showed that plateau pikas with bacterial enterotypes 1 and 3 had significantly higher body temperature and RMR than those with enterotype 2 (Fig. 7), indicating that pikas with the two enterotypes harbored higher energy metabolism than those with enterotype 2. Although pikas with enterotype 3 exhibited higher RMR than those with enterotype 2, both maintained their thermogenesis (equal to RMR) stably between warm and cold seasons (Fig. 8A). Thus, high energy expenditure during the cold season could significantly decrease body temperature (Fig. 8). Of course, to protect against cold environments, pikas can maintain body temperatures by huddling or timing their above-surface activity according to the ambient temperature (35, 69). Nearly all pikas with enterotype 1 disappeared in the cold season (Fig. 1E), which may suggest that enterotype 1 pikas cannot acclimatize to the cold season or transform into other enterotypes. In contrast, we observed a significant increase in the proportion of enterotype 2 pikas during the cold season (Fig. 1E). The opposite change may be attributable to variations in nutrition levels of food resources between the warm and cold seasons. For example, the proportion of crude fat in the vegetation significantly decreases during the cold season, which might be a disadvantage to high-fat diet-associated enterotype 1, whereas the crude fiber levels significantly increased in the cold season, which might be an advantage for the high-fiber diet of enterotypes 2 and 3 (10, 11). Many dominant bacteria in enterotypes 2 and 3 belong to cellulose degradation taxa, such as unclassified *Oscillospiraceae*, unclassified *Lachnospiraceae*, and *Prevotella* which can transform the fiber-rich diet to short-chain fatty acids (SFCAs) (10, 43, 70). SFCAs are important energy and signal molecules that regulate thermogenesis, which can improve host fitness in cold environments (20). Interestingly, we detected increased enterotype 1 and decreased enterotype 3 at high altitudes compared to low altitudes (Fig. 1E). Additionally, pikas with enterotype 1 had a higher body temperature and RMR at high altitudes (Fig. 8B). The crude fat and fatty acids of many herbages preferred by pikas significantly increased at high altitudes, which may be an advantage for pikas with high-fat diet enterotype 1, which can provide the host with energy to maintain high body temperatures to survive in cold environments.

Pikas with fungal enterotype 1 displayed higher body temperatures than those with fungal enterotype 2, while the RMR and RMR/Mass of the two enterotypes did not differ significantly (Fig. 7). Pikas with fungal enterotype 1 engaged in coprophagy, which can supplement their food intake, thus contributing to high body temperature (35). Additionally, the higher insulation property of pikas with fungal enterotype 1 compared to those with fungal enterotype 2 may explain why both groups exhibited equal RMR and RMR/Mass but displayed significantly distinct body temperatures (71). We found that pikas in both fungal enterotype groups adopted an energy-saving strategy during the cold season, as they exhibited significantly lower body temperature in the cold season than in the warm season (Fig. 8C). Interestingly, the two fungal enterotypes displayed distinct strategies in cold environments at high altitudes. Pikas with fungal enterotype 2 can resist cold environments by increasing thermogenesis to maintain high body temperature (Fig. 8D). This may be attributed to the fact that many fungal taxa can promote high-fat diet-induced obesity (40), which, in turn, promotes the relative abundance of fungal taxa with a higher capacity to harvest energy from the diet (23, 72). Indeed, we detected significantly higher body mass at high altitudes than at low altitudes for pikas with fungal enterotype 2 but not for those with fungal enterotype 1 (Fig. S7).

In conclusion, we identified three bacterial and two fungal enterotypes. The predominant microbial taxa, community diversity, microbial co-occurrence networks, community assembly processes, body temperature, and RMR were distinct between enterotypes. Bacterial enterotype 1 was a high-fat diet enterotype that was suitable during the warm season, while the other two enterotypes were high-fiber diet enterotypes, which were suitable during the cold season. Enterotypes 1 and 3 showed higher thermogenesis than enterotype 2. Bacterial taxa in enterotype 3 displayed higher phylogenetic clustering than those in enterotypes 1 and 3. The neutral process was more important in structuring the community in enterotype 3 than that in the other two enterotypes. Plateau pikas with fungal enterotype 1 were widely distributed at low altitudes and with higher body temperatures, while pikas with fungal enterotype 2 were more frequently detected at high altitudes and with low body temperatures. Fungal taxa in the two enterotypes displayed high phylogenetic randomization, and the neutral process was important for structuring the community assembly in the two enterotypes. Taken together, we found that enterotypes were closely associated with the host survival environment, and pikas with different enterotypes harbored distinct microbial community compositions and functions, body temperatures, and RMR, which can promote host acclimatization to harsh environments in the plateau.

## MATERIALS AND METHODS

### Samples

A total of 308 wild plateau pikas were captured from 10 different habitats over a 2-year period between August 2019 and January 2021 on the Qinghai-Tibet Plateau. The sample sites were Qingshizui, Haibei, Taxiuxiang, Reshui, Zeku, Maqin, Goulixiang, Wozhuoyi pass, Maduo, and Kunlun mountain passes with altitudes ranging from 3,118 to 4,761 m, latitudes ranging from 34.45 to 37.67 °N, and longitudes ranging from 94.07 to 101.66 °E. The samples collected in August and January represent warm and cold seasons, respectively. According to the physiological and reproductive characteristic of plateau pikas (73 – 76), we defined the samples collected over altitude 3,900 m as high altitudinal samples, while the samples collected below 3,900 m were defined as low altitudinal samples. On average, 10 individuals were randomly captured at each sampling site. The body temperature (rectal temperature) and mass were recorded. The RMR of all individuals was measured within 12 h of capture. Thereafter, pikas were euthanized and dissected. Cecal contents were collected and flash-frozen using liquid nitrogen or dry ice and then stored at –80°C until DNA extraction.

### Body temperature, body mass, and RMR

Body temperature was measured using an anal thermometer (±0.1°C), and body mass was measured using a spring balance (±0.2 g) after the experimental subjects were captured. RMR was measured as carbon dioxide production per hour per mine (mL CO_2_/min) using an 8-channel FMS (Sable Systems International, Henderson, NV, USA) portable respiratory metabolism system.

### DNA extraction and sequencing

Total DNA was extracted from 308 samples (approximately 10 individuals per treatment) using the E.Z.N.A. soil DNA Kit (Omega Bio-tek, Norcross, GA, USA) according to the manufacturer’s protocol. The DNA extract was analyzed on a 1% agarose gel, and DNA concentration and purity were determined using a NanoDrop 2000 UV-vis spectrophotometer (Thermo Scientific, Wilmington, USA). The hypervariable region V3-V4 of the bacterial 16S rRNA gene and *ITS* gene of fungi were amplified with primer pairs 338F (5′-ACTCCTACGGGAGGCAGCAG-3′) and 806R (5′-GGACTACHVGGGTWTCTAAT-3′) and (*ITS1F*: CTTGGTCATTTAGAGGAAGTAA; *ITS2R*: GCTGCGTTCTTCATCGATGC ) using an ABI GeneAmp 9700 PCR thermocycler (ABI, CA, USA). The PCR amplification of 16S rRNA gene was performed as follows: initial denaturation at 95°C for 3 min, followed by 27/35 cycles of denaturation at 95°C for 30 s, annealing at 55°C for 30 s, extension at 72°C for 45 s, single extension at 72°C for 10 min, and ending at 4°C.

PCR mixtures included 4 µL 5× Fast Pfu buffer, 2 µL 2.5 mM dNTPs, 0.8 µL of each primer (5 µM), 0.4 µL of Fast Pfu DNA polymerase, 10 ng of extracted DNA, and ddH_2_O to achieve a final volume of 20 µL. PCR was performed in triplicate. The PCR product was extracted from a 2% agarose gel, purified using the AxyPrep DNA Gel Extraction Kit (Axygen Biosciences, Union City, CA, USA) according to the manufacturer’s instructions, and quantified using a Quantus Fluorometer (Promega, USA). Purified amplicons were pooled in equimolar amounts and paired-end sequenced on an Illumina MiSeq PE300 platform (Illumina, San Diego, USA) according to the standard protocols of Majorbio Bio-Pharm Technology Co. Ltd. (Shanghai, China). Raw reads were deposited in the NCBI Sequence Read Archive database.

### Amplicon sequence processing and analysis

Sequencing results were demultiplexed, and the resulting sequences were quality filtered using fastp (0.19.6) and merged with FLASH (v1.2.11); high-quality sequences were de-noised using the DADA2 (Callahan et al., 2016) plugin in the QIIME 2 (version 2020.2) pipeline with recommended parameters, which obtains single-nucleotide resolution based on error profiles within samples. DADA2 de-noised sequences are known as amplicon sequence variants (ASVs). To minimize the effects of sequencing depth on alpha and beta diversity measurements, the number of sequences from each sample was rarified to the minimum sequences represented in the total samples, which yielded an average Good’s coverage of 99.93% and 99.99% for bacterial and fungal communities, respectively. Taxonomic assignment of ASVs was performed using the Naive Bayes consensus taxonomy classifier in QIIME 2 and the SILVA 16S rRNA database (v138) or UNITE 8.0. Analyses of 16S rRNA and ITS sequencing data were performed using the Majorbio Cloud Platform (cloud.majorbio.com).

### Functional pathways prediction by PICRUSt2

The functional pathways of the gut bacteria were predicted using PICRUSt2 (Phylogenetic Investigation of Communities by Reconstruction of Unobserved States) (77) based on the ASV table. The functional proﬁle of the genes was determined with the identiﬁcation in KEGG pathways (78). The functional prediction of fungi was based on FUNGuild database, which was used to group each ASV into different ecological guild (79). The number of ASVs indicated the richness of a guild, and the relative abundance of each guild was calculated by dividing the number of sequences of a specific guild by the total number of sequences (80).

### Enterotype clustering

The enterotype was identified at the genus level of the gut microbiotas according to the partition around medoids (PAM) algorithm (81) on Jensen–Shannon distances using the R package “cluster,” and the optimal numbers of the cluster was identified by the Calinski–Harabasz (CH) index (82) performing in the R package “clusterSim.” The k-means clustering algorithms implemented in R package “factoextra,” and the best clustering was identified by the total WSS and within groups sum of squares using elbow method (83). The genus with the high relative abundance in each enterotype and the abundance of the genus was also significantly higher than that in other enterotypes was considered the main contributor of each enterotype. Only two samples in the cold season were sorted into enterotype 1; thus, we ignored the analysis related to enterotype 1 in the warm season samples.

### Co‐occurrence network

Co-occurrence network analysis of ASVs with a total relative abundance over 0.5% was used to investigate the relationship of the microbial community within each enterotype. Each dot represents a bacterial or fungal taxon, and the links represent statistically significant (*P* < 0.01) correlations (Pearson’s correlation) among the taxa; the correlation coefficients were over |0.7| for bacterial taxa and |0.5| for fungal taxa. The number of nodes and edges, average degree, and modularity were calculated in R using the package “igraph.” The networks were visualized using the Gephi v0.9.2 software (84).

### Null model

The mean nearest taxon distance (MNTD) computes the minimum mean of phylogenetic distance between an ASV and all other ASVs in each community (65). Standardized effect size measure (ses.MNTD) values were used to assess the assembly processes of the community as a reflection of their phylogenetic relationships. An ses.MNTD value >+2 or <−2 indicated significant phylogenetic clustering or phylogenetic overdispersion, and both indicated that deterministic processes dominated the community assembly. The ses.MNTD value was between −2 and +2, indicating the coexisting taxa are phylogenetic random or stochastic processes governing community assembly.

### Neutral model

The Sloan neutral model (68) was applied to determine the importance of the neutral process in the microbial community assembly. In general, the model predicts that abundant taxa in the metacommunity disperse between communities mainly via chance, whereas rare taxa are more likely to become extinct in different communities due to ecological drift (68). In this model, Nm is the estimate of dispersal between communities. Parameter *R* ^2^ represents the degree of fit to the neutral model. A high *R* ^2^ value indicated a good fit to the neutral model or a high stochastic process assembly of the community assembly. In this study, data sets from each enterotype were used to fit the neutral model separately. The ASVs from each enterotype were subsequently separated into three partitions based on the 95% confidence interval of the neutral model prediction: above-neutral (ASVs occurred more frequently than those predicted by the neutral model), below-neutral (occurred less frequently than predicted), or neutral (within prediction).

### Data analysis

The alpha diversities of bacteria and fungi were calculated on a rarified data set using the QIIME2 pipeline. We used the Kruskal-Wallis and Mann-Whitney *U* tests to compare the differences in alpha diversities, relative abundance of predominant taxa, ses.MNTD values, host body temperature and mass, RMR, and per unit mass of RMR between enterotypes. The chi-square test was used to test the shifts in the proportion of each enterotype across the seasons and altitudes. Principal coordinate analyses based on Bray-Curtis distances were conducted to compare the differences in gut microbial communities among pikas between different enterotypes. Permutational multivariate analysis of variance (PERMANOVA) was used to test for significant differences in the microbial community structure between enterotypes. Linear discriminant analysis effect size (LEfSe) and Kruskal-Wallis were used to assess differences in functional pathways (based on the KEGG database for bacterial taxa and FUNGuild database for fungal taxa) between enterotypes.

## Data Availability

The sequence data were deposited in the Sequence Read Archive (SRA) at NCBI under the accession numbers SRP433096 for bacteria and SRP433075 for fungi.

## SUPPLEMENTAL MATERIAL

Fig. S1 spectrum.00020-23-s0001.pdf Identiﬁcation of bacterial and fungal enterotypes by k-means clustering algorithm in pika.

Fig. S2 spectrum.00020-23-s0002.pdf Characteristics of bacterial and fungal enterotypes associated with host sex.

Fig. S3 spectrum.00020-23-s0003.pdf The correlation between microbial taxa at genus level is estimated by Spearman's correlation analysis.

Fig. S4 spectrum.00020-23-s0004.pdf The association between bacterial and fungal enterotypes.

Fig. S5 spectrum.00020-23-s0005.pdf Comparison of the functional differences between enterotypes.

Fig. S6 spectrum.00020-23-s0006.pdf Comparison of host body mass between enterotypes for total, warm and cold season, and low- and high-altitude samples.

Fig. S7 spectrum.00020-23-s0007.pdf The effect of seasons and altitudes on host body mass within each enterotype.

Table S1 spectrum.00020-23-s0008.docx The relative abundance of bacterial genera that significantly correlated with fungal genera in warm and cold seasons, low and high altitudes, and total samples.

Table S2 spectrum.00020-23-s0009.docx The topological property of bacterial co-occurrence network.

Table S3 spectrum.00020-23-s0010.docx The topological property of fungal co-occurrence network.

ASM does not own the copyrights to Supplemental Material that may be linked to, or accessed through, an article. The authors have granted ASM a non-exclusive, world-wide license to publish the Supplemental Material files. Please contact the corresponding author directly for reuse.
