# Supplementary material for: Temporal and geographic distribution of gut microbial enterotypes associated with host thermogenesis characteristics in plateau pikas
Source: Microbiol Spectr. 2023 Oct 10;11(6):e00020-23. doi: 10.1128/spectrum.00020-23 (PMC10715161; doi:10.1128/spectrum.00020-23)
Supplement: Fig. S1 — Identiﬁcation of bacterial and fungal enterotypes by k-means clustering algorithm in pika. [file spectrum.00020-23-s0001.pdf]

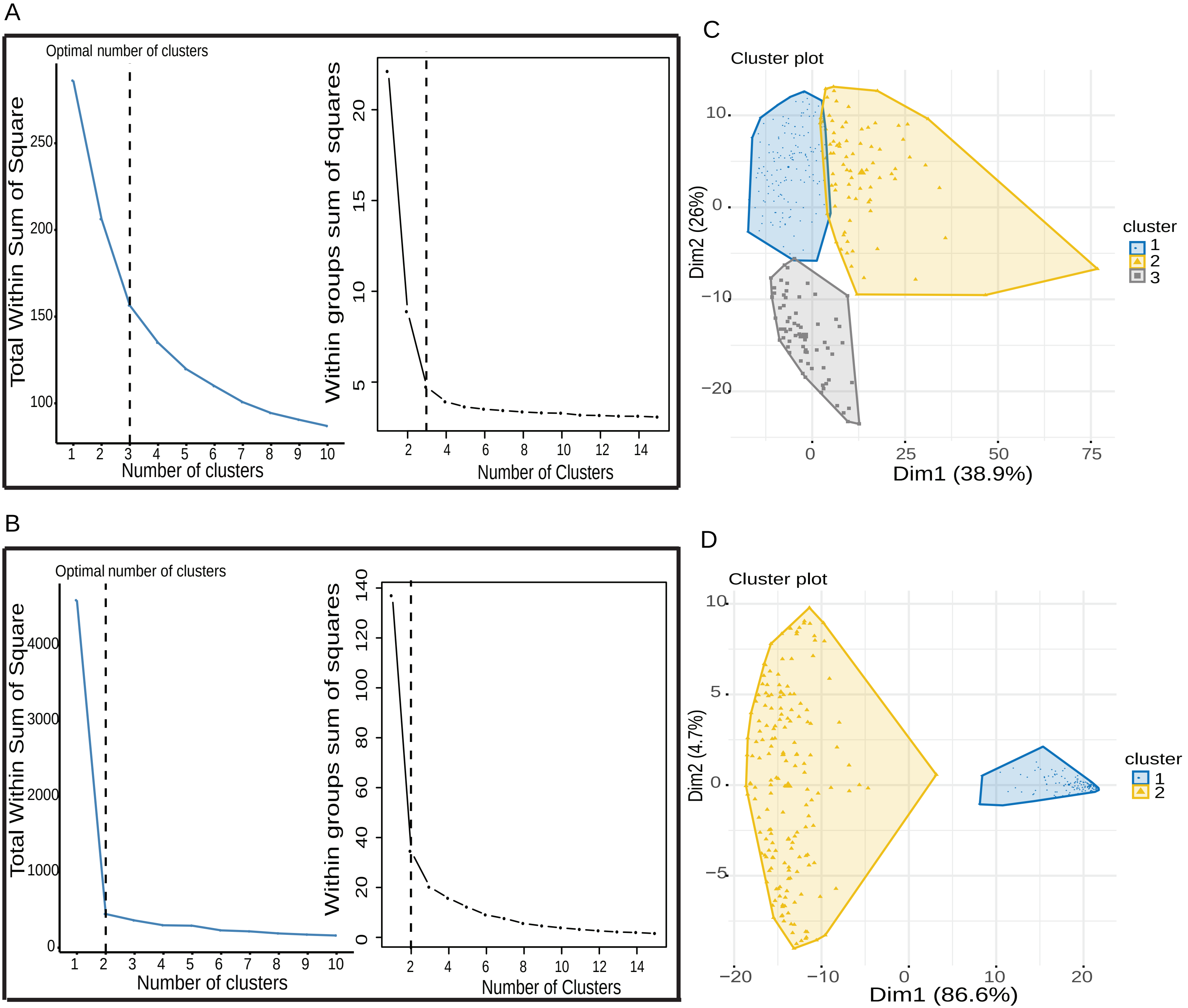

Figure S1 Identification of bacterial and fungal enterotypes by k-means clustering algorithm in pika. (A) The optimal number of clusters for bacterial community based on total within-cluster sum of squares and within groups sum of squares using elbow method. (B) The optimal number of clusters for fungal community based on total within-cluster sum of squares and within groups sum of squares using elbow methods. (C) The principal component analysis (PCA) based on Jensen–Shannon distance for bacterial community. (D) The principal component analysis (PCA) based on Jensen–Shannon distance for fungal community.
