# Supplementary material for: Temporal and geographic distribution of gut microbial enterotypes associated with host thermogenesis characteristics in plateau pikas
Source: Microbiol Spectr. 2023 Oct 10;11(6):e00020-23. doi: 10.1128/spectrum.00020-23 (PMC10715161; doi:10.1128/spectrum.00020-23)
Supplement: Fig. S2 — Characteristics of bacterial and fungal enterotypes associated with host sex. [file spectrum.00020-23-s0002.pdf]

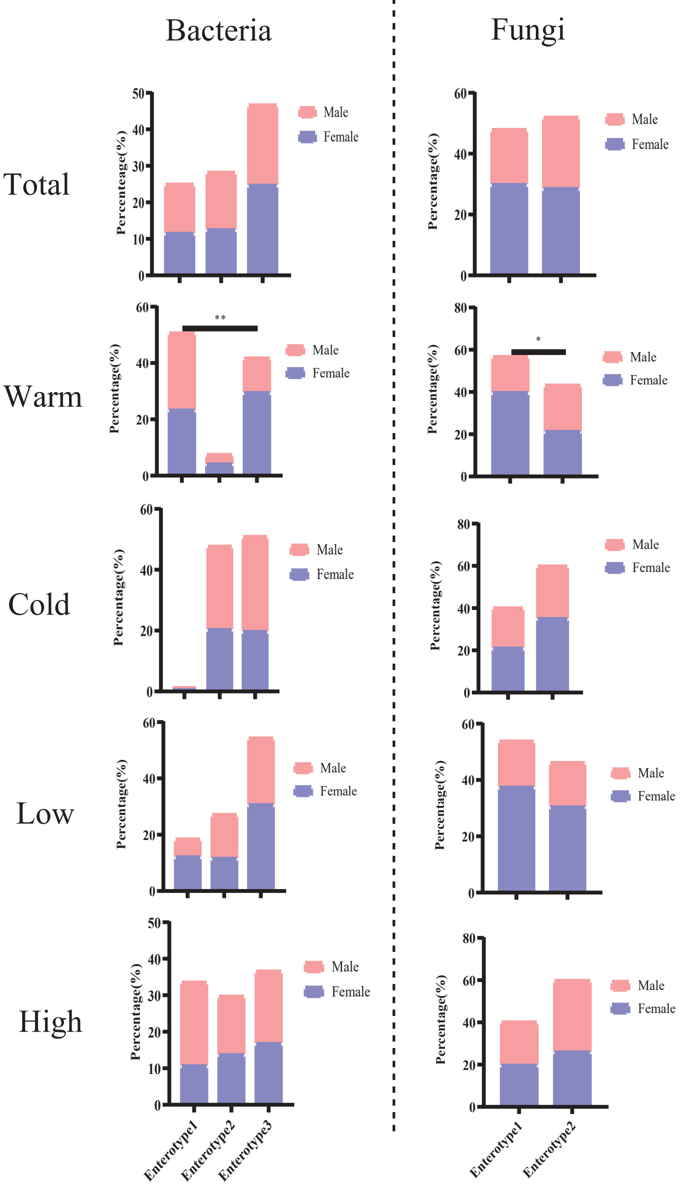

Figure S2 Characteristics of bacterial and fungal enterotypes associated with host sex. “Total” represent total sample; “Warm” and “Cold” represent samples from warm and cold seasons, respectivly; “Low” and “High” represent samples from low and high altitudes, respectivly.
