# Supplementary material for: Temporal and geographic distribution of gut microbial enterotypes associated with host thermogenesis characteristics in plateau pikas
Source: Microbiol Spectr. 2023 Oct 10;11(6):e00020-23. doi: 10.1128/spectrum.00020-23 (PMC10715161; doi:10.1128/spectrum.00020-23)
Supplement: Fig. S3 — The correlation between microbial taxa at genus level is estimated by Spearman's correlation analysis. [file spectrum.00020-23-s0003.pdf]

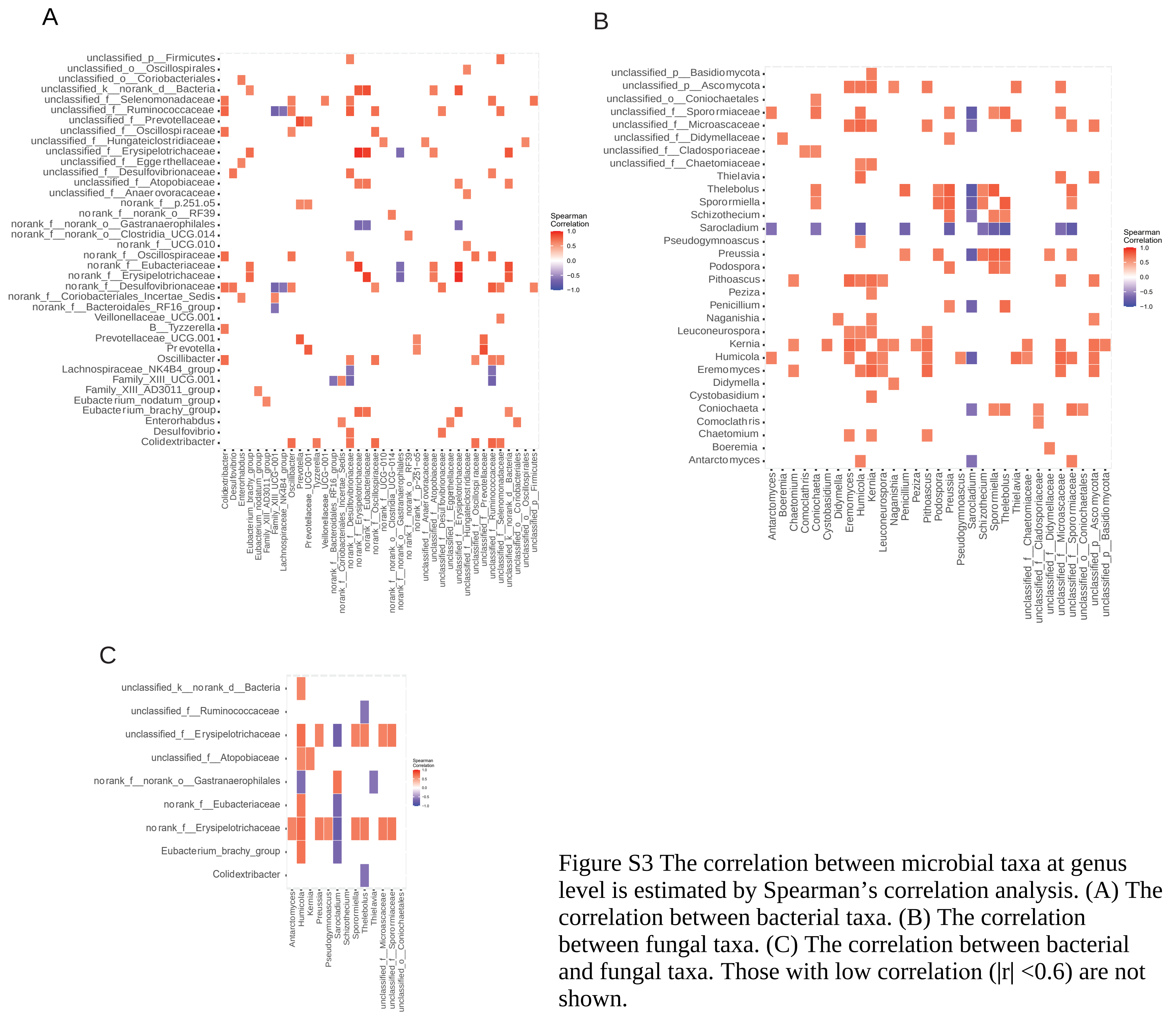

Figure S3 The correlation between microbial taxa at genus level is estimated by Spearman's correlation analysis. (A) The correlation between bacterial taxa. (B) The correlation between fungal taxa. (C) The correlation between bacterial and fungal taxa. Those with low correlation (|r| <0.6) are not shown.
