# Supplementary material for: Temporal and geographic distribution of gut microbial enterotypes associated with host thermogenesis characteristics in plateau pikas
Source: Microbiol Spectr. 2023 Oct 10;11(6):e00020-23. doi: 10.1128/spectrum.00020-23 (PMC10715161; doi:10.1128/spectrum.00020-23)
Supplement: Fig. S4 — The association between bacterial and fungal enterotypes. [file spectrum.00020-23-s0004.pdf]

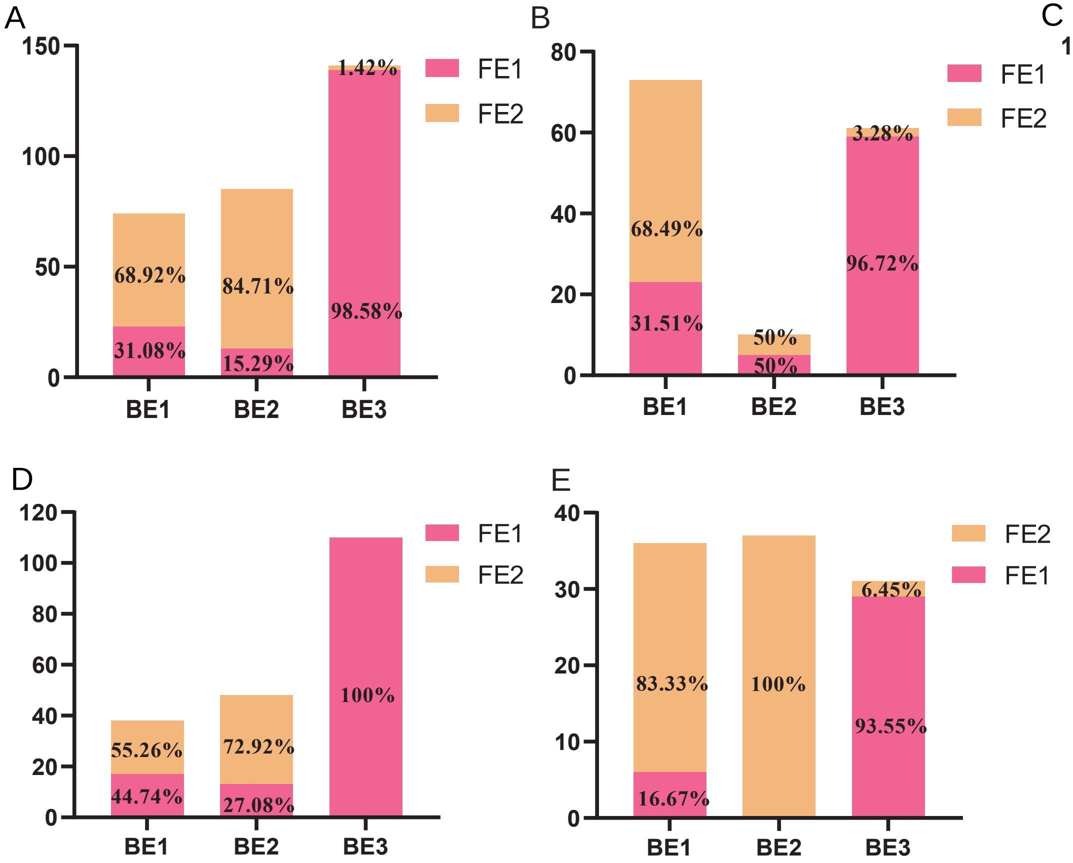

Figure S4 The association between bacterial and fungal enterotypes. The proportion of fungal enterotypes in each bacterial enterotype for the total (A), warm (B) and cold (C) seasons, low (D) and high (E) altitudes samples. The BE1, 2 and 3 represent bacterial enterotype 1, 2 and 3, rspectively. The FE1 and 2 represent fungal enterotype 1 and 2, respectively.
