# Supplementary material for: Temporal and geographic distribution of gut microbial enterotypes associated with host thermogenesis characteristics in plateau pikas
Source: Microbiol Spectr. 2023 Oct 10;11(6):e00020-23. doi: 10.1128/spectrum.00020-23 (PMC10715161; doi:10.1128/spectrum.00020-23)
Supplement: Fig. S5 — Comparison of the functional differences between enterotypes. [file spectrum.00020-23-s0005.pdf]

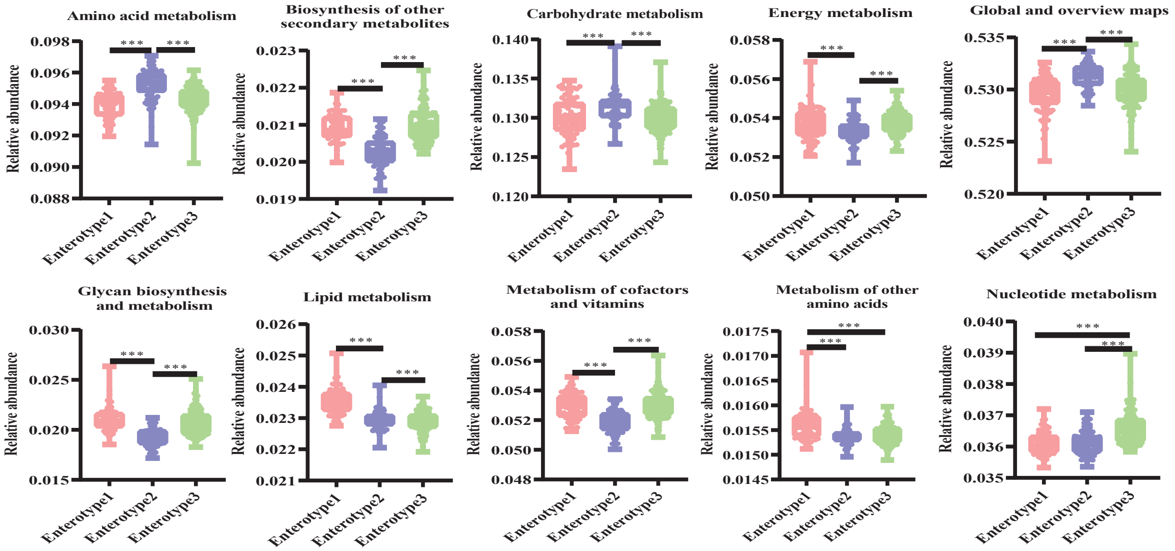

Figure S5 Comparison of the functional differences between enterotypes. The top ten abundant bacterial functional pathways at level two were compared using the Kruskal–Wallis test. The asterisks indicate * $p < 0.05$, ** $p < 0.01$, *** $p < 0.001$.
