# Supplementary material for: Temporal and geographic distribution of gut microbial enterotypes associated with host thermogenesis characteristics in plateau pikas
Source: Microbiol Spectr. 2023 Oct 10;11(6):e00020-23. doi: 10.1128/spectrum.00020-23 (PMC10715161; doi:10.1128/spectrum.00020-23)
Supplement: Fig. S6 — Comparison of host body mass between enterotypes for total, warm and cold season, and low- and high-altitude samples. [file spectrum.00020-23-s0006.pdf]

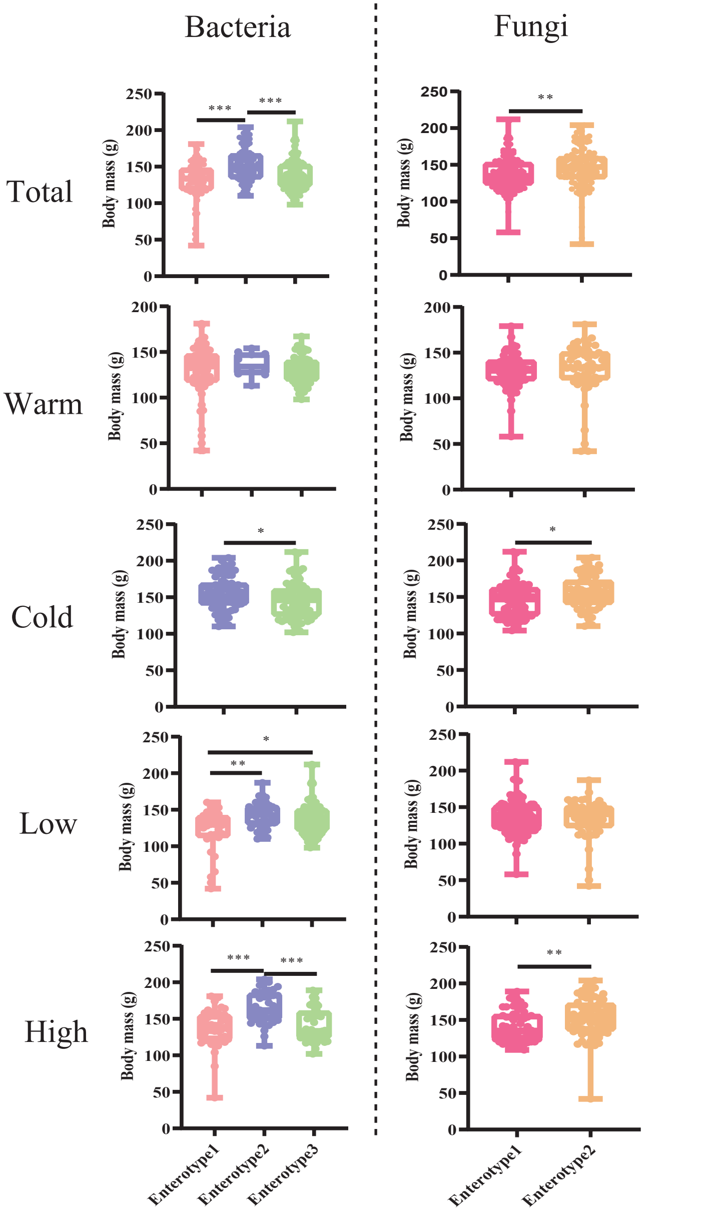

Figure S6. Comparison of host body mass between enterotypes for total, warm and cold seasons, and low- and high-altitudes samples. The asterisks indicate * $p < 0.05$, ** $p < 0.01$, *** $p < 0.001$ (Mann–Whitney U and Kruskal–Wallis tests).
