# Supplementary material for: Temporal and geographic distribution of gut microbial enterotypes associated with host thermogenesis characteristics in plateau pikas
Source: Microbiol Spectr. 2023 Oct 10;11(6):e00020-23. doi: 10.1128/spectrum.00020-23 (PMC10715161; doi:10.1128/spectrum.00020-23)
Supplement: Fig. S7 — The effect of seasons and altitudes on host body mass within each enterotype. [file spectrum.00020-23-s0007.pdf]

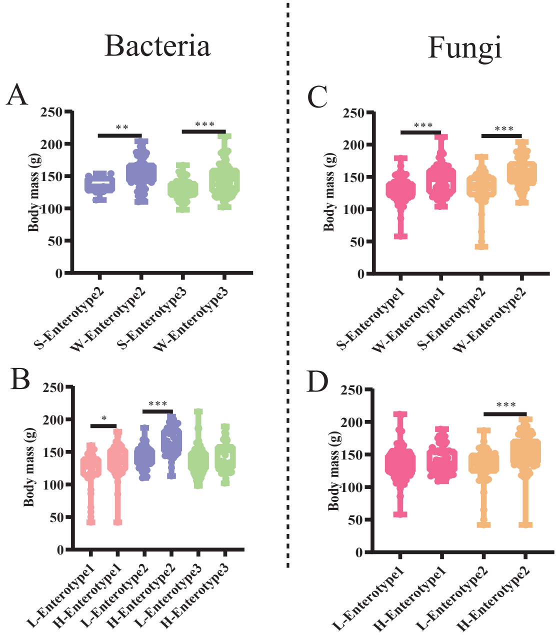

Figure S7 The effect of seasons and altitudes on host body mass within each enterotype. Comparison host body mass between warm and cold seasons (a and c) and low and high altitudes (b and d) within each enterotype. The asterisks indicate * $p < 0.05$, ** $p < 0.01$, *** $p < 0.001$ (Mann–Whitney U and Kruskal–Wallis tests).
