# Supplementary material for: Temporal and geographic distribution of gut microbial enterotypes associated with host thermogenesis characteristics in plateau pikas
Source: Microbiol Spectr. 2023 Oct 10;11(6):e00020-23. doi: 10.1128/spectrum.00020-23 (PMC10715161; doi:10.1128/spectrum.00020-23)
Supplement: Table S1 — The relative abundance of bacterial genera that significantly correlated with fungal genera in warm and cold seasons, low and high altitudes, and total samples. [file spectrum.00020-23-s0008.docx]

Table S1 The relative abundance of bacterial genera that significant correlated with fungal genera (Fig. S 3C) in warm and cold seasons, low and high altitudes, and total samples.

| Genus | Warm season | cold season | low altitude | high altitude | total |
| --- | --- | --- | --- | --- | --- |
| Colidextribacter | 0.004 | 0.010 | 0.006 | 0.010 | 0.007 |
| unclassified_f_Ruminococcaceae | 0.002 | 0.006 | 0.003 | 0.006 | 0.004 |
| norank_f_Erysipelotrichaceae | 0.003 | 0.003 | 0.003 | 0.003 | 0.003 |
| unclassified_f_Erysipelotrichaceae | 0.003 | 0.003 | 0.003 | 0.002 | 0.003 |
| norank_f_Eubacteriaceae | 0.001 | 0.002 | 0.002 | 0.001 | 0.002 |
| unclassified_k_norank_d_Bacteria | < 0.001 | 0.001 | 0.001 | < 0.001 | 0.001 |
| norank_f_norank_o_Gastranaerophilales | < 0.001 | 0.001 | < 0.001 | 0.001 | < 0.001 |
| unclassified_f_Atopobiaceae | < 0.001 | < 0.001 | < 0.001 | < 0.001 | < 0.001 |
| Eubacterium_brachy_group | < 0.001 | < 0.001 | < 0.001 | < 0.001 | < 0.001 |
