# Supplementary material for: Temporal and geographic distribution of gut microbial enterotypes associated with host thermogenesis characteristics in plateau pikas
Source: Microbiol Spectr. 2023 Oct 10;11(6):e00020-23. doi: 10.1128/spectrum.00020-23 (PMC10715161; doi:10.1128/spectrum.00020-23)
Supplement: Table S2 — The topological property of bacterial co-occurrence network. [file spectrum.00020-23-s0009.docx]

Table S2 The topological property of bacterial co-occurrence network

|  | Total nodes | Total edges | Positive edges | Negative edges | Average degree | Modularity |
| --- | --- | --- | --- | --- | --- | --- |
| **Total samples** | | | | | | |
| Enterotype1 | 171 | 146 | 145 | 1 | 1.708 | 0.953 |
| Enterotype2 | 240 | 266 | 264 | 2 | 2.217 | 0.905 |
| Enterotype3 | 98 | 134 | 133 | 1 | 2.735 | 0.724 |
| **Warm season** | | | | | | |
| Enterotype1 | 174 | 149 | 148 | 1 | 1.713 | 0.955 |
| Enterotype2 | 464 | 2066 | 1432 | 634 | 8.905 | 0.575 |
| Enterotype3 | 119 | 174 | 171 | 3 | 2.924 | 0.659 |
| **Cold season** | | | | | | |
| Enterotype2 | 168 | 162 | 161 | 1 | 1.929 | 0.947 |
| Enterotype3 | 76 | 100 | 99 | 1 | 2.632 | 0.786 |
| **Low altitude** | | | | | | |
| Enterotype1 | 305 | 253 | 249 | 4 | 1.659 | 0.962 |
| Enterotype2 | 289 | 407 | 404 | 3 | 2.817 | 0.855 |
| Enterotype3 | 125 | 186 | 176 | 10 | 2.976 | 0.722 |
| **High altitude** | | | | | | |
| Enterotype1 | 207 | 223 | 222 | 1 | 2.155 | 0.887 |
| Enterotype2 | 271 | 357 | 350 | 7 | 2.635 | 0.854 |
| Enterotype3 | 207 | 250 | 249 | 1 | 2.415 | 0.853 |
