# Supplementary material for: Temporal and geographic distribution of gut microbial enterotypes associated with host thermogenesis characteristics in plateau pikas
Source: Microbiol Spectr. 2023 Oct 10;11(6):e00020-23. doi: 10.1128/spectrum.00020-23 (PMC10715161; doi:10.1128/spectrum.00020-23)
Supplement: Table S3 — The topological property of fungal co-occurrence network. [file spectrum.00020-23-s0010.docx]

Table S3 The topological property of fungal co-occurrence network

|  | Total nodes | Total edges | Positive edges | Negative edges | Average degree | Modularity |
| --- | --- | --- | --- | --- | --- | --- |
| **Total samples** | | | | | | |
| Enterotype1 | 628 | 1804 | 1788 | 16 | 5.745 | 0.898 |
| Enterotype2 | 462 | 3122 | 3122 | 0 | 13.515 | 0.691 |
| **Warm season** | | | | | | |
| Enterotype1 | 460 | 1485 | 1479 | 6 | 6.457 | 0.832 |
| Enterotype2 | 333 | 2226 | 2226 | 0 | 13.369 | 0.644 |
| **Cold season** | | | | | | |
| Enterotype1 | 428 | 1817 | 1743 | 74 | 8.491 | 0.783 |
| Enterotype2 | 178 | 1536 | 1536 | 0 | 17.258 | 0.444 |
| **Low altitude** | | | | | | |
| Enterotype1 | 472 | 1657 | 1649 | 8 | 7.021 | 0.835 |
| Enterotype2 | 215 | 1255 | 1255 | 0 | 11.674 | 0.622 |
| **High altitude** | | | | | | |
| Enterotype1 | 394 | 2883 | 2779 | 104 | 14.635 | 0.556 |
| Enterotype2 | 242 | 1548 | 1548 | 0 | 12.793 | 0.678 |
